# scExtract: leveraging large language models for fully automated single-cell RNA-seq data annotation and prior-informed multi-dataset integration

**DOI:** 10.1186/s13059-025-03639-x

**Published:** 2025-06-19

**Authors:** Yuxuan Wu, Fuchou Tang

**Affiliations:** 1 Biomedical Pioneering Innovation Center, School of Life Sciences, Peking University, Beijing, 100871 China; 2 Beijing Advanced Innovation Center for Genomics (ICG), Ministry of Education Key Laboratory of Cell Proliferation and Differentiation, Beijing, China

**Keywords:** Single-cell RNA sequencing, Large language models, Dataset integration

## Abstract

Single-cell RNA sequencing has revolutionized cellular heterogeneity research, but analyzing the abundance of unannotated public datasets remains challenging. We present scExtract, a framework leveraging large language models to automate scRNA-seq data analysis from preprocessing to annotation and integration. scExtract extracts information from research articles to guide data processing, outperforming existing reference transfer methods in benchmarks. We introduce scanorama-prior and cellhint-prior, which incorporate prior annotation information for improved batch correction while preserving biological diversities. We demonstrate scExtract’s utility by integrating 14 datasets to create a comprehensive human skin atlas of 440,000 cells.

**Supplementary Information:**

The online version contains supplementary material available at 10.1186/s13059-025-03639-x.

## Background

Since the advent of single-cell RNA sequencing technology [1], facilitated by breakthroughs in experimental procedures and sequencing platforms, the growth of publicly available single-cell sequencing data has continuously expanded very rapidly. Decreasing costs and widespread adoption of commercialized protocols, such as the 10X Genomics platform, have made single-cell RNA sequencing ubiquitous across biological disciplines. To mitigate resource wastage from redundant sequencing, third-party public datasets have become indispensable for research discovery and validation. Consequently, large-scale, curated single-cell “atlas” datasets have emerged for critical and complex diseases [2].

**Fig. 1:**
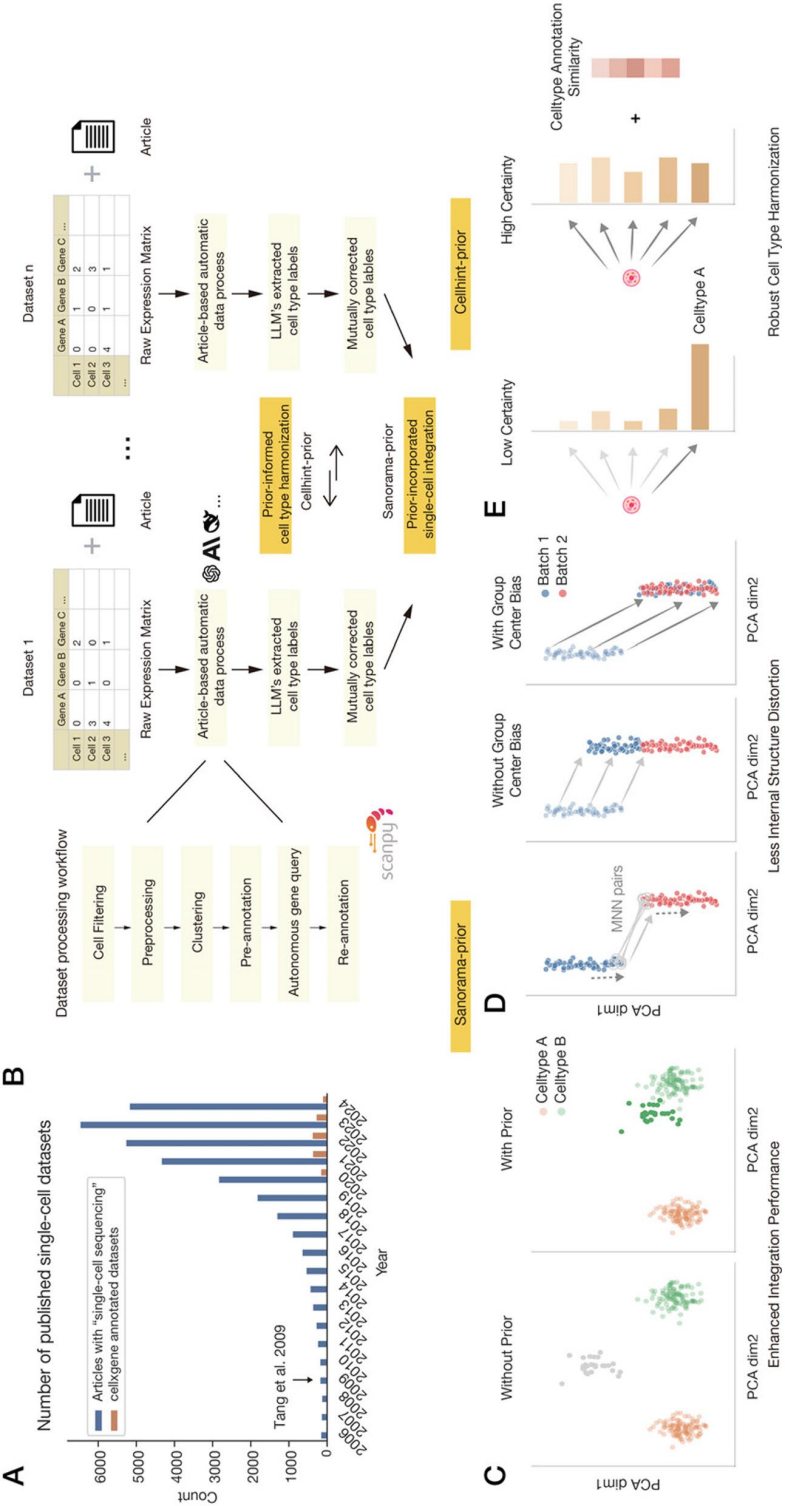
Workflow of scExtract. **A** Bar chart comparing the annual number of newly published single-cell sequencing articles versus datasets available on crowdsourcing platforms. **B** Schematic workflow illustrating the integration and annotation steps of scExtract. Individual datasets are first processed separately by the LLM agent, including preprocessing and annotation, before being merged together using annotation-based prior information for integration. **C** Scatter plot showing that Scanorama-prior utilizes label prior information to facilitate integration. **D** Scatter plot showing that Scanorama-prior better preserves the internal structure of cell clusters during integration. Left panel: During integration, Scanorama calculates mutual nearest pairs between two batches and determines the integration displacement vector (light gray arrows) through mutual nearest neighbor pairs. Scanorama-prior builds upon this by additionally considering the relative positions of cells within the clusters (gray dotted arrows). Middle panel: In Scanorama integration, cells move according to the original light gray displacement vectors, causing all Batch 1 cells to shift to the upper portion of Batch 2. Right panel: Scanorama-prior considers relative positions, using gray arrows for displacement, resulting in better preservation of internal structure. **E** Cellhint-prior dynamically adjusts the weights of prior knowledge based on the uncertainty of cell population assignments. When uncertainty is higher during the integration process, cellhint-prior incorporates a greater proportion of prior knowledge, ensuring the cautious introduction of prior information

Collaborative efforts like the Human Cell Atlas (HCA) [3] and crowdsourcing platform such as the cellxgene [4] platform have driven the generation of extensive, multi-species, cross-tissue, and multi-omics cellular datasets. As of August 9, 2024, cellxgene, the largest literature-curated single-cell database, encompasses 1458 datasets, primarily human and mouse single-cell RNA sequencing data. However, this progress is overshadowed by the annual influx of thousands of publications with novel single-cell sequencing datasets (Fig. 1A). These discrete datasets span fundamental cellular processes to diverse human pathologies, including cancer, neurodegenerative disorders, and immunological conditions. Effective utilization of these resources could enhance understanding from common to rare diseases with diverse cellular states while facilitating the construction of a comprehensive, multi-dimensional cellular landscape across a wide variety of tissues, developmental time points, diseases, and treatment conditions, thereby reducing research inefficiencies.

Current data sharing protocols only mandate submission of raw sequencing data, without requiring processed expression matrices. While some publications provide cell-level annotations based on cellular barcodes in supplementary materials, these are often already incorporated into public datasets. Many smaller-scale published datasets frequently lack accessible single-cell level annotations. This deficiency creates a significant barrier for data integration, necessitating time-consuming manual review of articles to extract preprocessing methodologies and marker gene descriptions before dataset processing—an approach that becomes untenable when constructing large-scale integrated datasets from literature.

Automated annotation of single-cell datasets using reference label transfer methods offers an alternative strategy [5]. However, this approach limits the discovery of novel cell types unique to smaller datasets and requires comprehensive, high-quality reference labels, often unavailable for many disease subtypes. Another method involves integrating unlabeled datasets using batch correction algorithms [6], followed by unified clustering and annotation. Nevertheless, existing batch correction methods tend to favor predominant cell types, potentially over-integrating dataset-specific rare cell populations.

Recent advancements in natural language processing and general-purpose large language models (LLMs) have expanded their applications to extracting structured biological knowledge. Cell annotation methods utilizing LLMs like GPT-4 have shown impressive performance in evaluations [7]. However, applying these methods to automatically process article data presents several challenges. These include conducting cell filtration and preprocessing based on article information, determining optimal clustering granularity that aligns with the authors’ biological understanding, and leveraging article-derived prior information for marker gene-based cell type annotation. Incorporating this contextual knowledge is likely to yield superior performance compared to approaches that do not consider such article-specific information.

In light of these characteristics, we have developed scExtract, a novel framework based on large language models for fully automated extraction and integration of published single-cell RNA-seq data using an annotation-aware approach. scExtract requires only raw expression matrices and article content as input, automatically performing preprocessing, clustering, and annotation operations aligned with the original methods described in the articles. Furthermore, scExtract enables integration in a prior-dependent manner, utilizing previous automated annotations with modified versions of scanorama [8] and cellhint [9], which outperform original methods that do not incorporate prior knowledge across various metrics.

In articles with well-annotated datasets, scExtract demonstrates higher accuracy surpassing current established methods across tissues. We further leveraged the advantages of annotation by developing scExtract’s integration pipeline, which incorporates annotations as prior knowledge. Through comparative analyses across multiple datasets of varying scales, we demonstrated that scExtract’s integration method not only exhibited enhanced batch correction results while preserving biological diversities, but also demonstrated robust performance even with some ambiguous or erroneous labels. By combining annotation and integration capabilities, scExtract enables rapid and accurate integration of published single-cell omics datasets.

We applied this comprehensive pipeline to 14 skin scRNA-seq datasets encompassing various conditions, automatically constructing a skin immune dysregulation dataset comprising over 440,000 cells. Analysis of this integrated dataset validated different activation program of T helper cells across various diseases. Detailed subgroup analysis revealed characteristic cell cluster expansion of proliferating keratinocytes in psoriasis, one of the most prevalent autoimmune skin disorders. These findings highlight the utility and effectiveness of scExtract framework in uncovering novel biological insights from diverse single-cell omics sequencing datasets.

## Results

### Article-based automated dataset process of scExtract

scExtract’s automated processing pipeline consists of two components: LLM-based automatic annotation incorporating article background information, and cell-type harmonization with embedding integration guided by annotation information (Fig. 1B). In the annotation phase, we implemented an LLM agent that emulates human expert analysis, automatically processing datasets while incorporating article background information. For the integration phase, to optimize the annotated data processing, we utilized the preliminary annotation information. We modified two well-performing software packages, scanorama and cellhint, and integrated them in a pipeline that leverages approximate annotations to enhance dataset integration.

In annotation stage, scExtract employs scanpy [10], the standard Python framework for single-cell data analysis, to perform computations. The standard processing pipeline includes cell filtering, preprocessing, unsupervised clustering, and cell population annotation. scExtract emulates the workflow adopted by human researchers in actual single-cell data annotation (Fig. 1B) (Methods), by extracting parameters used in each step from the article text and implementing them using the scanpy system. For instance, if the target article mentions in the “Methods” section, “We filtered out cells with ≥ 20% mitochondrial genes,” scExtract would extract this parameter and execute the corresponding computation.

In the clustering phase, scExtract’s prompts include two aspects: it can extract the number of cluster groups from the article as an external parameter, and when the article does not explicitly state the number of groups, it can infer from the article’s content, such as the number of cell populations discussed or the granularity of annotations. This method leverages the authors’ prior knowledge, which is crucial for preserving maximal biological significance in the clustering process. While algorithms exist for selecting optimal cluster numbers in unsupervised clustering, they are often not applied in practical data analysis due to their potential unreliability in capturing biologically meaningful distinctions.

During the annotation phase, similar to previous work utilizing LLMs for cell annotation, scExtract takes marker gene lists for each group as input. However, it also incorporates the article’s background knowledge, ensuring that annotation results align more closely with the article’s content. To better mitigate hallucinations in LLM information extraction and differences in the implementation platform of processing workflows, scExtract can optimize previous annotations after the initial annotation by autonomously querying the expression levels of a set of characteristic marker genes. This gene set is generated by scExtract based on the article and existing tissue and cell type information, inferring potential cell populations in the current microenvironment and characteristic genes of annotated but low-confidence groups. After obtaining the expression levels of these genes across different groups, scExtract can further optimize its previous annotations.

The next step in constructing a fully automated dataset is to integrate the data annotated by scExtract. However, the majority of conventional integration methods fail to leverage prior annotation information. To overcome these limitations, we developed scanorama-prior. Scanorama-prior requires additional clustering information and a similarity matrix of cell annotations at the embedding level, both from the scExtract processing pipeline. When constructing mutual nearest neighbors (MNN), scanorama-prior considers the prior differences between cell types (Fig. 1C), adjusting the weighted distances between cells across datasets, thereby achieving more accurate neighbor construction.

Additionally, when shifting cells between datasets, scanorama-prior tends to move original cell groups as cohesive units towards corresponding groups in the target dataset. To enhance batch correction, it applies an additional adjustment vector based on the positions of cell group centers in both datasets. The weight of this adjustment vector is determined by the annotation similarity between the original and target groups. Consequently, if two groups exhibit similar expression patterns and identical annotations, they will be more uniformly integrated (Fig. 1D). This approach ensures that biological relationships are preserved while effectively mitigating batch effects.

While possessing various advantages, scanorama-prior exhibits sensitivity to annotation errors, a characteristic we detailed in the benchmark section. To address this limitation, we incorporated cellhint-prior into scExtract’s integration method. Cellhint, originally designed for cluster-level integration, serves as an ideal downstream complement to rough annotations. Cellhint’s methodology can be employed to rectify previous annotations and provide harmonized annotations through cross-dataset comparisons. To mitigate potential interference from prior information, we adopted a conservative approach in incorporating prior knowledge by adjusting annotation weights based on cell alignment uncertainty levels (Fig. 1E)(Methods).

scExtract implements a stepwise integration process for automatically annotated datasets (Fig. 1B). The process begins with cellhint-prior for cell type harmonization, leveraging neighboring datasets as references to rectify potential nomenclature inconsistencies stemming from LLM output variations. When embedding-level integration is necessary, scanorama-prior is applied to the harmonized cell types. The text-to-embedding approach’s flexibility in cell type input format enables the similarity matrix to be derived from harmonized cell types. This pipeline effectively addresses both annotation harmonization and embedding integration challenges in heterogeneous single-cell omics datasets.

### Evaluating clustering and annotation accuracy using cellxgene data

**Fig. 2:**
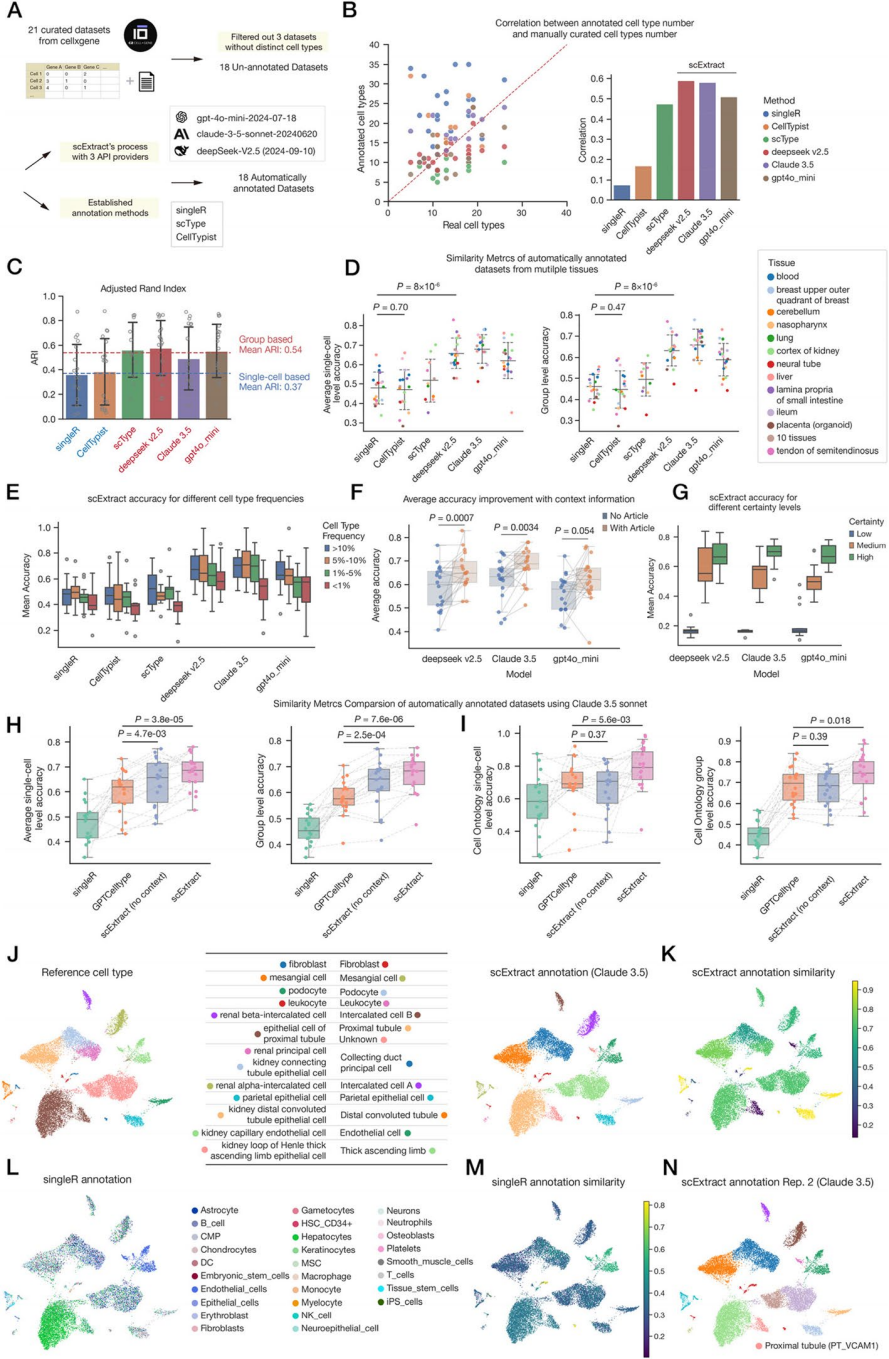
scExtract automatically annotates published datasets with high accuracy. **A** Schematic workflow of the cellxgene datasets benchmark annotation task. Raw expression matrices from 18 cellxgene datasets were downloaded, processed through scExtract using three model provider APIs, and were benchmarked against curated annotations. **B** Dot plot showing the correlation between true cell types and automatically annotated cell types. Correlation coefficient calculated using Pearson’s correlation. **C** Bar plot showing the adjusted rand index between curated and annotated groups. **D** Dot plot illustrating the comparison of cell annotation similarity metrics on benchmark datasets, using text-to-embedding similarity. The left side shows the accuracy averaged at the single-cell level, while the right side shows the accuracy averaged at the group level. *p*-values calculated using Wilcoxon signed-rank test. **E** Box plot demonstrating the relationship between cell type annotation accuracy and data abundance across different cell populations. **F** Box plot comparing the improvement in annotation accuracy when incorporating literature knowledge versus direct annotation. *p*-values calculated using Wilcoxon signed-rank test. **G** Box plot showing the relationships between confidence levels assigned by scExtract using different LLMs and actual annotation accuracy. **H,I** Dot plot illustrating the comparison of cell annotation similarity metrics across four different annotation methods on benchmark datasets with Claude 3.5 sonnet as the annotation model. **H** shows text-to-embedding similarity measurements, while **I** presents cell-ontology similarity metrics. Within each panel, the left side displays cell-level average accuracy, while the right side shows cluster-level accuracy. *p*-values calculated using Wilcoxon signed-rank test. **J** UMAP plot displaying standard cell types in a kidney dataset (left) and cell types automatically annotated by scExtract using Claude 3.5 (right). **K** UMAP visualization demonstrating the text-to-embedding accuracy of cell type annotations generated by scExtract. **L** UMAP visualization showing cell type annotations generated by SingleR. **M** UMAP visualization illustrating the text-to-embedding accuracy of SingleR annotations. **N** UMAP visualization showing cell type annotations from a second independent run of scExtract using Claude 3.5 Sonnet, aligned with literature-defined cell types

We utilized manually annotated datasets from cellxgene to assess annotation accuracy. We randomly selected 21 medium-scale annotated datasets (on the order of 10^4^ cells), 18 of which possessed diverse cell types (Fig. 2A, Additional file 2: Table S1) [11–27]. These datasets encompass samples from multiple human tissues or organs, including liver, kidney, and intestine, addressing a wide range of distinct biological contexts. We compared scExtract with three established methods, including SingleR [28], scType [29], and CellTypist [30] (Methods). To ensure cost-effectiveness when applied to larger-scale data, we employed three model providers supportive of large-scale queries, with longer context (> 128 k tokens) and suitable pricing (≤ $5.00 per 1 M input tokens): Deepseek-v2.5 [31], GPT-4o-mini [32], and Claude-3.5-sonnet [33].

We first evaluated the preservation of group structure in single-cell RNA-seq datasets across different automatic annotation methods. Regarding the discrepancy between annotated cell type numbers and those reported in the original articles, SingleR and CellTypist, which operate at the single-cell annotation level, exhibited a clear tendency to overestimate cell type numbers due to single-cell level noise. In contrast, scType and scExtract, which utilize marker gene lists for cell group annotation, demonstrated better correlation with curated cell type numbers (Fig. 2B). Among all model providers used in scExtract, Claude 3.5 tends to overestimate the number of cell types, while Deepseek-v2.5 and GPT-4o-mini tend to underestimate the number of cell types. We further assessed the clustering performance using the Adjusted Rand Index (ARI) (Fig. 2C). Group-level annotation methods demonstrated significantly higher structural similarity compared to single-cell-level approaches. Meanwhile, Claude 3.5, due to its propensity for detailed annotation, displayed the lowest ARI among all models.

To evaluate annotation accuracy, we employed large language model’s text-to-embedding method, converting the annotation information into embeddings and calculating cosine similarity (Methods). scExtract-based methods demonstrated higher mean accuracy than current established methods across all 18 datasets in text-to-embedding (Fig. 2D). Moreover, reference transfer methods are more likely to accurately capture common cell types comprising the majority of the dataset due to their frequent occurrence in reference datasets. We observed that the annotation accuracy of all methods decreased as the cell proportion in the overall dataset declined (Fig. 2E). To address this limitation of single-cell level average, we implemented a cluster-level accuracy metric that disregards the influence of cell numbers. This metric better reflects the accuracy of automatic annotation for rare cell types. Using this metric, scExtract methods showed a clear advantage (Fig. 2D). The performance differences within various language models is less pronounced, with Claude 3.5 demonstrating the most accurate information extraction capability, followed closely by Deepseek v2.5, and then GPT-4o-mini, which is roughly consistent with these models’ performance in other domains. Additionally, we also evaluated the relationships between standard and annotated cell types using Cell-Ontology label (Methods). The results were consistent with embedding-based methods, with a more pronounced disparity between group-level and single-cell-level approaches in reference transfer methods (Additional file 1: Fig. S1A).

Compared to the previously published GPTCelltype [7] method, we hypothesize that scExtract’s superior annotation accuracy stems from its application of article-derived prior knowledge and self-reflective capability through two-round annotation. During the annotating step, scExtract completed its annotations based on background and characteristic genes, then reviewed and adjusted the annotation content. For instance, in the annotation of actual datasets, during the second round of annotation, scExtract subdivided intercalated cells into two subgroups by querying the expression levels of *SLC26A7* and *SLC26A4* in the second round (Additional file 1: Fig. S1B-D).

We evaluated the performance difference between scExtract with and without background knowledge integration (Fig. 2F). Methods incorporating background knowledge demonstrated improved accuracy, and we hypothesize that the varying degrees of improvement among different models depend on their ability to capture contextual information from the background text. We also observed that as text was proportionally replaced with confounding contents, annotation accuracy declined to varying degrees, although more sophisticated models like Claude3.5 exhibited a comparatively smaller decrease in performance (Additional file 1: Fig. S2A-B).

We directly compared the accuracy of GPTCelltype and scExtract methods on our dataset across different models and metrics, with scExtract consistently demonstrating significant improvements than GPTCelltype (Fig. 2H,I, Additional file 1: Fig. S2C-D). When assessing annotation accuracy using text-to-embedding methodology, the performance hierarchy was clearly established: scExtract > scExtract (no context) > GPTCelltype. Similarly, in cell ontology-based accuracy measurements, the performance ranking was as follows: scExtract > scExtract (no context) ≈ GPTCelltype. Notably, the performance differences between scExtract (no context) and GPTCelltype varied according to the specific accuracy metric employed. We attribute these variations to inherent methodological differences in the evaluation approaches. The text-to-embedding method may be more susceptible to formatting inconsistencies, particularly regarding standardized Cell Ontology annotation usage, whereas the Ontology Look Service (ols_api) effectively neutralized these differences by systematically mapping annotations to the standardized Cell Ontology hierarchical structure.

To mitigate the black-box nature of LLMs, we also implemented confidence scoring during the annotation process (Fig. 2G). Results validated that the confidence scores provided by scExtract reliably correlate with actual annotation accuracy.

### scExtract accurately annotated datasets with accordant group structure

Taking the scExtract-generated dataset of adult human kidney [15] annotated by Claude 3.5 as a representative example (Fig. 2J). The structure largely aligns with the clustering in the original article, demonstrating accuracy in annotation granularity. This contrasts with reference transfer-based methods, which often misclassified single cell types into heterogeneous populations due to expression fluctuations (Fig. 2L). Regarding annotation accuracy, most cell clusters were annotated consistently with the original definitions.

However, a small portion of cells showed lower accuracy (Fig. 2K), typically author-defined cell subtypes more susceptible to language model fluctuations. For instance cells annotated as “Unknown” by scExtract (Fig. 2J) were originally defined as PT_VCAM1 [15] (subpopulation of proximal tubule with *VCAM1* expression). Interestingly, this subtype was correctly annotated as “Proximal tubule (PT_VCAM1)” in the second replicates, matched to the original article (Fig. 2N, Additional file 1: Fig. S1D). Among model providers, Claude 3.5’s annotations adhered more closely to the original text, while Deepseek v2.5 and GPT-4o-mini tended to reduce the specificity of original expressions, providing more generic annotations (Additional file 1: Fig. S3A). These observations underscore the necessity for further refinement and validation of LLM-based annotations.

We also evaluated the poorer-performing neural tube dataset (Fig. 2D, Additional file 2: Table S1 dataset 4). Results indicated that scExtract’s lower performance largely stemmed from overly broad cell type definitions in the standard dataset, with more accurate cell type annotations in the original article (Additional file 1: Fig. S3B). For example, scExtract’s automatic annotations of two import structures, roof and floor plate cells, aligned well with marker gene expression patterns (Additional file 1: Fig. S3D). However, as clustering complexity and customization increased, annotations required more careful consideration.

Overall, scExtract maintained granularity and annotation fidelity while operating fully automatically. It can process novel datasets cost-effectively without relying on pre-annotated reference datasets, offering significant advantages in scalability and extensibility. Notably, scExtract can complete all process procedure of a dataset in less than 20 min (Additional file 1: Fig. S3E) at a cost of less than one dollar, with no additional computational resources required if using web API. Moreover, with the ongoing advancements in language models, we anticipate further improvements in precision and efficiency.

### Evaluating scExtract’s output stability and prompt sensitivity

Major constraints limiting the applications of large language models in scientific domains include output variability and susceptibility to prompt interference. We next assessed the performance of the LLM agent in scExtract with respect to these aspects. We selected Sample1, Sample11, and Sample19 (Additional file 2: Table S1), repeating the analysis of each sample for six times to evaluate performance across two task phases: parameter extraction during preprocessing and cell annotation labeling.

For preprocessing parameters, high consistency was observed when parameters were explicitly stated in the manuscripts (Additional file 1: Fig. S4A-B). Variability occurred primarily for non-critical parameters not explicitly defined, where the LLM made reasoned inferences and cautiously set them to “default” rather than fabricating values. Reduced reproducibility was noted in complex scenarios such as manuscripts containing multiple datasets with different characteristics, unpublished manuscripts, or custom algorithms (Additional file 1: Fig. S4C). For cell annotations, high reproducibility was achieved for most cell types across replicates. Variability was typically confined to cell subpopulations with ambiguous marker profiles (Additional file 1: Fig. S5A). Solid tissue samples (Samples 11, 19) (Additional file 1: Fig. S7A-B) showed higher annotation consistency than blood samples (Sample 1) (Additional file 1: Fig. S5A). Annotation variability correlated with cell population heterogeneities and was accurately reflected in the model’s self-reported certainty levels (Additional file 1: Fig. S7A, S8B). When comparing different methods, we observed that strict reproducibility ranked as GPTCelltype > scExtract (no context) > scExtract (Additional file 1: Fig. S5A-B, S6A). However, this ranking inversely correlated with annotation specificity, as more detailed annotations naturally introduced more potential variations. Methods lacking article context failed to identify novel cell subtypes discussed in the original papers (Additional file 1: Fig. S9A-B).

We tested prompt sensitivity using Sample 11 with five prompt variants: instruction sequence reordering, terminology modification with synonyms, enhanced guidance with detailed instructions, minimalistic annotation with minimal instructions, and structural reformatting using JSON output format (Methods). Results showed that parameter extraction was generally robust across prompt variations. Claude 3.5 Sonnet maintained more consistent performance with simplified prompts compared to Deepseek v3 (Additional file 1: Fig. S10A-B). Similarly, annotation content remained largely stable across prompt variants, particularly for well-defined cell types (Additional file 1: Fig. S11A-B) (Additional file 3: Table S2). Advanced models demonstrated reduced sensitivity to prompt variations (Additional file 1: Fig. S11A-B).

In conclusion, scExtract maintains robust performance across multiple replicates and prompt variations, particularly for well-defined parameters and cell types. Variability occurs primarily in scenarios requiring nuanced interpretations of ambiguous data. The implementation of certainty reporting and detailed reasoning logs provides users with transparency regarding the model’s decision-making process, enabling appropriate interpretation of the results.

### Scanorama-prior enhanced integration of annotated single-cell omics datasets

**Fig. 3:**
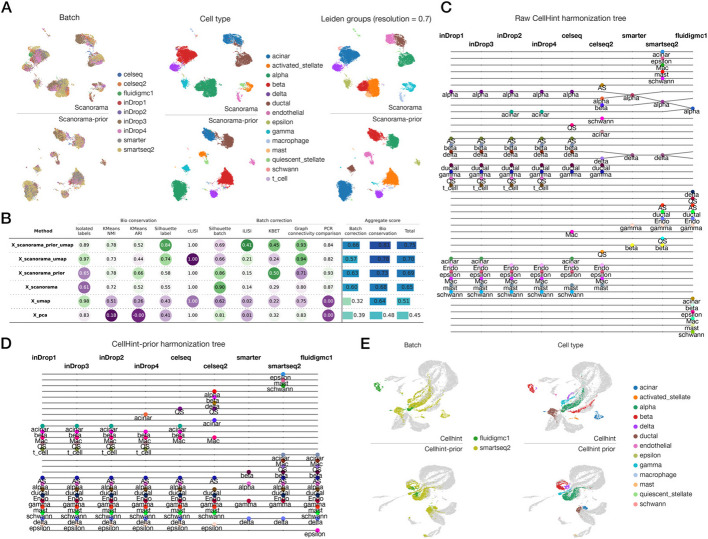
Prior incorporated integration method benchmark of human pancreas dataset. **A** UMAP plot comparing the integration performance of Scanorama (upper panels) and Scanorama-prior (lower panels) on pancreatic datasets. Each column from left to right shows batch, cell type, and the results of unsupervised clustering using the integrated embedding. **B** Table presenting the batch correction and biological conservation performance of Scanorama, Scanorama-prior, and unintegrated datasets. Overall aggregate results are displayed in the horizontal barplot on the right. **C,D** Tree plots illustrating the performance of the original Cellhint (**C**) and Cellhint-prior (**D**) on pancreatic data. Each column represents a batch, with dots representing cell type groups within the batch. Horizontal lines between dots indicate they were aligned across datasets. **E** UMAP plot demonstrating the integration of pancreatic data in UMAP dimensions using Cellhint (upper panels) and Cellhint-prior (lower panels)

We evaluated scanorama-prior’s integration performance using pancreas single-cell RNA-seq datasets, which utilized different sequencing platforms on pancreas samples [34]. In contrast to the original version’s integration results, scanorama-prior demonstrated superior batch effect removal while preserving cell type differences (Fig. 3A). Utilizing identical resolution parameters, unsupervised clustering on the adjusted embeddings revealed that scanorama’s results displayed more intra-cluster separations resembling batch distributions, primarily due to incomplete batch effect removal. We also benchmarked corrected PCA and UMAP embeddings on batch correction and biological diversity preservation [6]. Results showed that scanorama-prior’s embeddings in both spaces exhibited superior performance metrics compared to the original version (Fig. 3B).

To evaluate scanorama-prior’s robustness to incorrect labeling, we tested its integration performance under three scenarios: similar naming (cell types replaced with aliases or alternative nomenclature), unbiased incorrect naming (erroneous naming without bias towards existing cell types, e.g., annotated as unknown), and biased incorrect naming (erroneous naming biased towards existing cell types, e.g., mislabeling one cell type as another). Our findings revealed that scanorama-prior performed well in the first two scenarios. In the first case, the integration efficacy was essentially indistinguishable from that achieved with strictly identical string inputs (Additional file 1: Fig. S12A). In the second scenario, the method adopted a more conservative approach to integrate the two cell types, yet still managed to merge them based on expression patterns (Additional file 1: Fig. S12B). However, in the third scenario, scanorama-prior received conflicting directives from two dimensions. Consequently, it refrained from integrating the cells into either group, instead positioning them as outliers between the two clusters (Additional file 1: Fig. S12C). This observation underscores the importance of ensuring basic similarity in annotation types when integrating datasets, or at the very least, avoiding biased incorrect annotations.

### Cellhint-prior enhanced cross-dataset annotation harmonization with improved tolerance for naming errors

To address this challenge in fully automate context, we leveraged a prior-aware version of cellhint, which can automatically reconcile annotation discrepancies across datasets. Evaluation using pancreas datasets revealed that while the original cellhint excelled in integrating inDrop series data, it struggled with Smart-seq2 and Fluidigm C1 platforms (Fig. 3C). In contrast, the prior-informed cellhint demonstrated superior alignment compared to its predecessor. Notably, it successfully aligned cells with severe batch effects due to disparities in batch and dataset origins (Fig. 3D, E). This improvement underscores the value of incorporating annotation priors in the cell type alignment process, particularly when dealing with heterogeneous data sources. We further evaluated cellhint-prior’s performance under those three previously mentioned annotation error scenarios. Results demonstrated that the structure of the cell type harmonization tree remained consistent with that of correct annotations across all three conditions (Additional file 1: Fig. S12F). This stability underscores the method’s enhanced robustness to annotation variations.

### scExtract’s two-step automated integration showed robust performance on mis-annotated cell types

Regarding the previously mentioned annotation error tolerance, we conducted multiple experiments to evaluate the impact of incorrect annotations on the scExtract two-step integration pipeline (Methods). In each experiment, we implemented all three types of label changes and isolated the modified data for subsequent benchmark evaluation (Additional file 1: Fig. S12A-C). Our assessment of integration performance metrics revealed that, consistent with previous benchmark results, scanorama-prior with prior knowledge performed optimally before label changes, while the use of cellhint for harmonization showed no significant impact (Additional file 1: Fig.S13A). However, after label changes, scanorama-prior without cellhint-prior harmonization showed marked performance degradation, while the scExtract’s prior-harmonized version maintained robust performance (Additional file 1:Fig.S13A).

Additionally, we performed unsupervised clustering on the post-change embeddings and annotated each cell cluster using majority voting. In scenarios where label changes resulted in cells being separately clustered, as in previous experiments, these cells would be misclassified. We observed that scanorama-prior-harmonized demonstrated the highest tolerance to annotation errors (Additional file 1: Fig. S13B-F), in some cases surpassing the tolerance of cellhint raw without prior knowledge integration. This pattern was also evident at the embedding level (Additional file 1: Fig.S14A-E). Scanorama-prior, obtained through our two-step integration approach, demonstrates optimal merging between cells with altered type labels (dark labels) and other cells of the same type (light background). This superior performance is attributed to two key factors: enhanced batch effect correction, compared to cellhint, and improved resilience to annotation variations, compared to un-harmonized scanorama-prior.

### Comprehensive evaluation of large-scale atlas integration demonstrates the scalable superiority of scExtract-based methods

We evaluated time performance of our two-step automated integration method on concatenated large datasets (Methods). scanorama-prior, which incorporates a prior similarity matrix, demonstrated slightly higher time complexity compared to the original scanorama method (Additional file 1: Fig. S15A) in a PC-level processer. To overcome the computational limitations of scanorama-based methods on large datasets, we implemented GPU acceleration. When executed on a V100 GPU, scanorama-prior exhibited speed increase compared to the original no-prior method and demonstrated scalability to datasets with one million cells (Additional file 1: Fig. S15B). Notably, in our benchmark tests, cellhint-based methods exhibited significantly longer execution time compared to those reported in the original paper [9] (Additional file 1: Fig. S15B). This discrepancy arises from our approach of generating large datasets by concatenating smaller ones, where the original dataset size increases proportionally with cell numbers, which is more reasonable in a real scenario. In contrast, benchmark in the original study employed downsampling from large datasets, maintaining constant original dataset numbers. We observed substantial runtime differences between these two approaches. However, since our raw datasets were relatively small, this represents an extreme case demonstration. In practice, actual execution times are expected to fall between these two extremes.

**Fig. 4:**
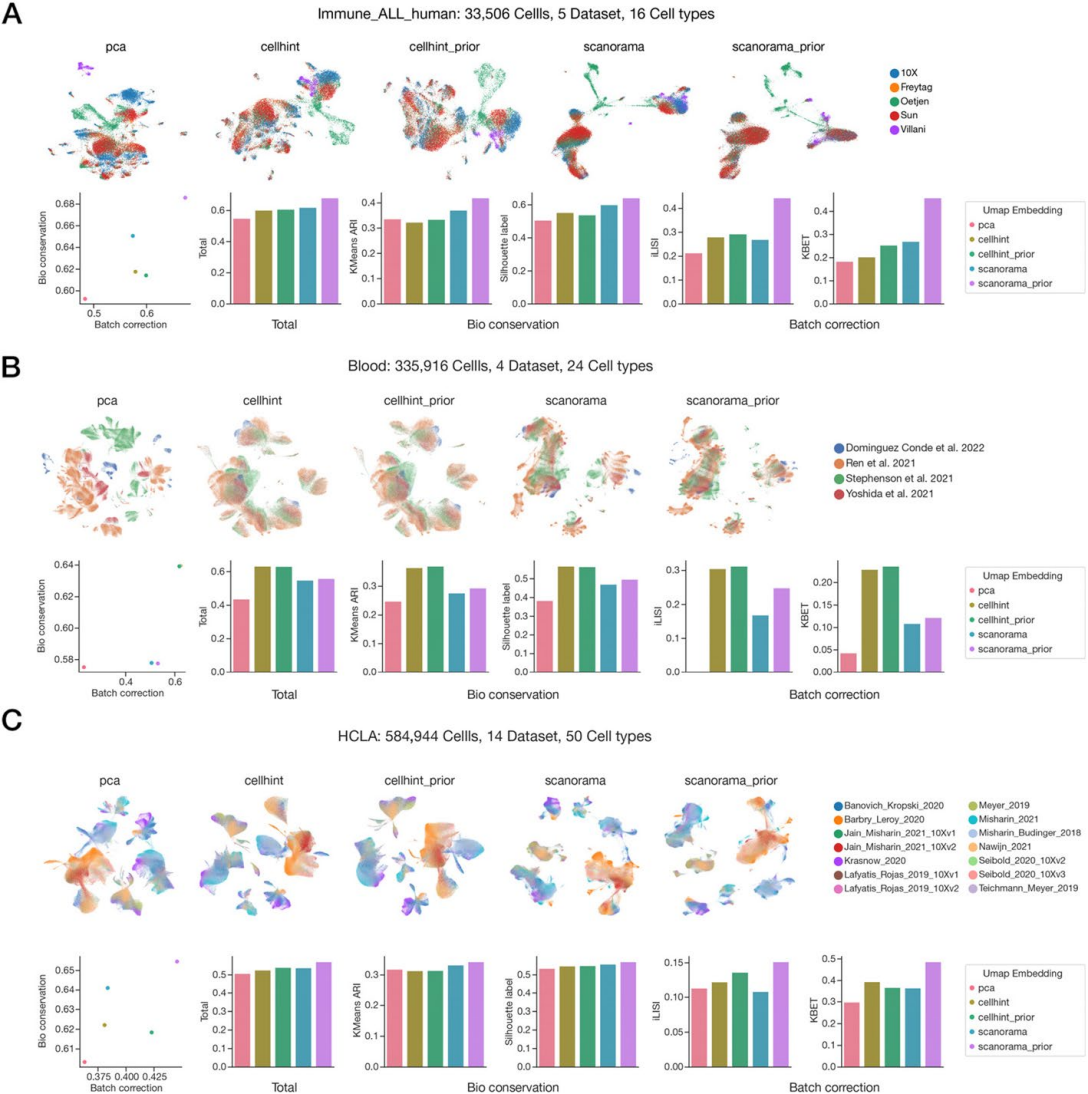
Assessment of scExtract’s two-step integration strategy on large-scale datasets. **A–C** Systematic evaluation of integration performance on Immune cells (**A**), Blood cells (**B**), and Human Lung Cell Atlas (**C**) datasets. Dataset statistics (number of cells, datasets, and cell types) are indicated above each panel. Upper panel: UMAP visualizations of embeddings generated by different methods (from left to right): PCA, cellhint integration, cellhint_prior integration, original scanorama, and scExtract’s two-step integration derived scanorama_prior. Lower panel: Performance metrics (from left to right): scatterplot showing batch effect removal versus biological variation preservation, barplot of comprehensive performance metrics, barplots displaying KMeans Adjusted Rand Index (ARI), Silhouette label scores, integration Local Inverse Simpson’s Index (iLISI), and *k*-nearest neighbor Batch Effect Test (kBET)

We further evaluated scExtract’s integration performance on six larger datasets [6, 9, 35], containing between 50,000 and 600,000 cells in total, representing typical real-world integration scenarios. We first tested the impact of adding general cell type descriptions to the integration process. Interestingly, we found virtually no differences in similarity matrices generated using either “cell type” alone or “cell type: description” (Additional file 1: Fig. S16A-B). This suggests that standard cell type designations already serve as sufficient sources of prior information. As cellhint’s integration approach modifies cell adjacency relationships rather than directly altering embeddings in reduced dimensional space, we generated 2D UMAP embeddings using identical parameters for benchmarking. In datasets around 50,000 cells level (Fig. 4A, Additional file 1: Fig. S17A), scanorama-prior, derived from scExtract’s two-step pipeline, demonstrated superior batch correction and biological preservation compared to both the original scanorama and other methods. This was followed by scanorama itself, with cellhint-prior and cellhint showing relatively poor performance. While we anticipated that direct embedding displacement would be more effective than graph relationship modification for smaller datasets, we hypothesize that single-cell noise might compromise the effectiveness of such approaches in larger-scale datasets.

For datasets containing more than 200,000 cells, we observed substantial variations in method performance across different datasets. In the Blood and Spleen datasets, containing 200,000 and 330,000 cells respectively but only four datasets each, benchmark results indicated that cellhint-prior and cellhint significantly outperformed other methods, with minimal performance differences between them (Fig. 4B, S17B). The original scanorama showed relatively poor performance, consistent with benchmark results reported in the corresponding literature. However, in the Human Oral Cell Atlas and Human Lung Cell Atlas examples, which comprised 250,000 cells/14 datasets and 580,000 cells/14 datasets, scanorama-prior, derived from our two-step integration, regained the leading position, while prior-knowledge-enhanced methods demonstrated increasingly larger advantages over their original counterparts (Fig. 4C, S17C).

Based on these results, we hypothesize that high-cell-count, low-dataset-number configurations may compromise the performance of embedding-based methods while favoring graph-construction approaches. From a practical perspective, we recommend terminating the integration process at the cellhint-prior step when the average cell number per dataset exceeds 50,000 or when dealing with fewer than four datasets, as this approach may yield optimal results. However, this modification does not diminish the significance of prior annotation, as both cellhint and cellhint-prior require pre-clustered data input.

Additionally, for novel datasets without reference materials, we tested first using scExtract (no context) to annotate the datasets, and then integrating them using scExtract’s two-step approach. We conducted analyses using pancreatic datasets as examples and performed three replicates. The overall performance of scExtract (no context) fell between that of scanorama-prior using standard cell types and the original scanorama (Additional file 1: Fig. S18). These results are reasonable from our perspective. When examining specific performance aspects, the batch correction effect of scExtract (no context) integration was significantly better than the original scanorama, approaching the level of scanorama-prior that directly used standard cell types for integration. However, in terms of biological preservation, scExtract (no context) showed a decline, likely due to potential annotation errors interfering with the integration steps. Notably, across different replicates, the composite scores remained highly robust (Additional file 1: Fig. S18), consistent with our previous conclusions from the annotation evaluation section.

### Automated construction of a skin autoimmune disease dataset with custom data incorporation

The skin serves as a critical barrier for our body, comprising various cell types. It is crucial for skin health to maintain homeostasis within its microenvironment [36]. Autoimmune conditions like psoriasis and atopic dermatitis (AD) often result from dysregulated micro-environmental signaling pathways involving complex interactions among keratinocytes, immune cells, fibroblasts, and other cellular components [37, 38]. Despite recently emerging large-scale datasets [39, 40], they often encompass limited disease phenotypes and require substantial resources for de novo single-cell sequencing. We utilized scExtract to automatically integrate skin datasets, addressing these limitations.

**Fig. 5:**
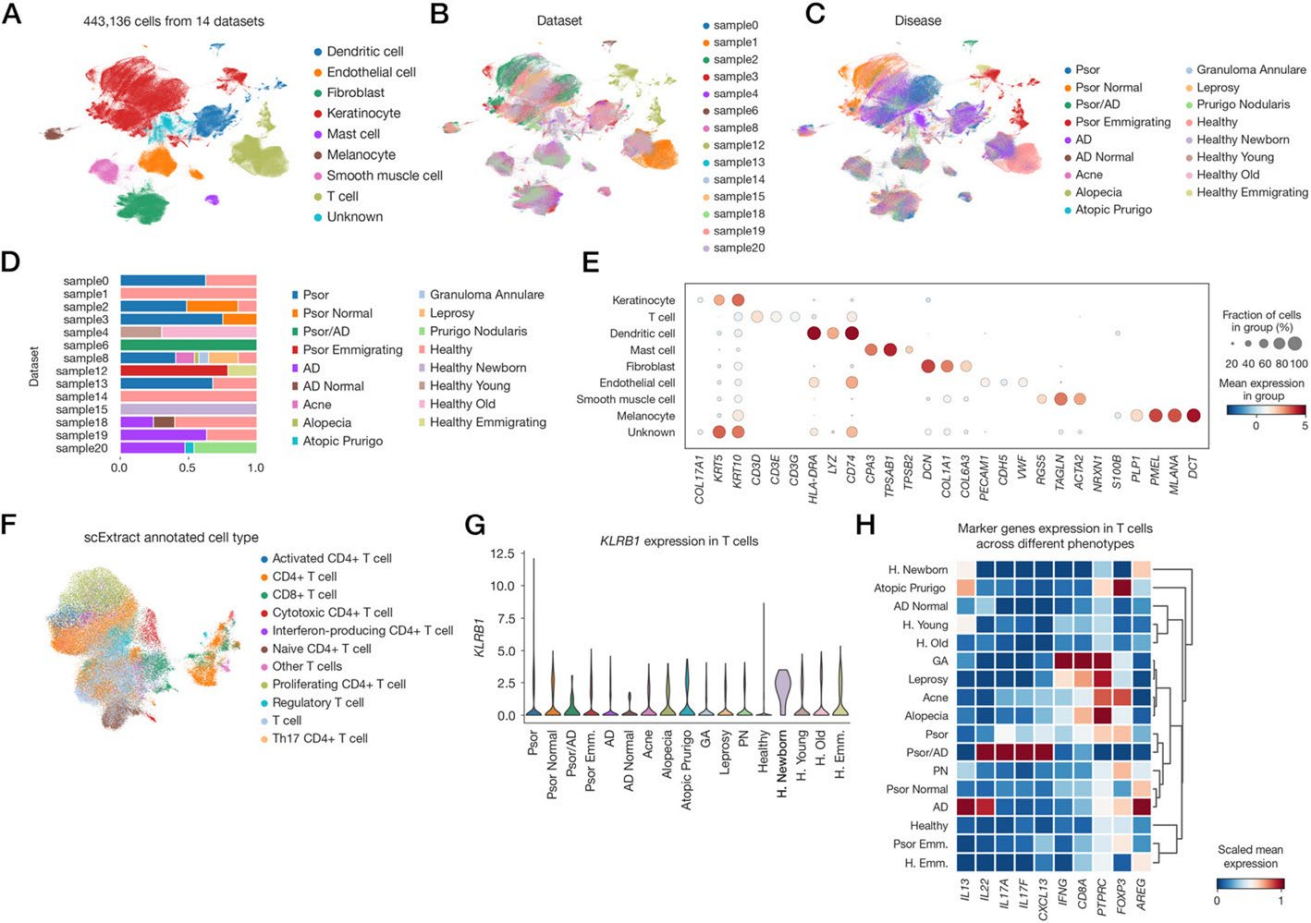
Analysis of scExtract generated human skin single-cell RNA-seq dataset. **A–C** UMAP plots displaying major voting annotation (**A**), dataset sources (**B**), and sample disease states and age labels (**C**) of automatically integrated datasets. **D** Horizontally normalized bar plot showing label distribution proportions across original datasets. **E** Dot plot illustrating marker gene expression levels across different cell types in automatically integrated datasets. **F** UMAP plot showing T cell subpopulation annotations generated by scExtract using Claude 3.5. **G** Violin plot demonstrating *KLRB1* expression levels as an innate lymphoid cells (ILCs) marker across different phenotypes in keratinocyte subpopulations. **H** Matrix plot depicting scaled expression levels of marker genes across different phenotypes in keratinocyte subpopulations

We screened 20 articles for dataset construction (Additional file 4: Table S3), excluding six due to species mismatch or inconvenient accession of raw data. Phenotype labels were manually curated from NCBI information, with acceleration of scExtract by extracting data accession and metadata of samples. Standard automated integration procedures using Claude 3.5 yielded a comprehensive dataset of 440,000 cells from the remaining 14 articles (Fig. 5A, B) [41–54]. This integrated dataset encompasses various pathologies including psoriasis, AD, acne, and granuloma annulare (GA), as well as across developmental stages from neonates to elderly individuals (Fig. 5C, D).

To validate dataset quality, we performed manual clustering using two-step scanorama-prior embedding (Additional file 1: Fig. S19A). To facilitate the annotation of integrated data, scExtract provides an automated fuzzy majority voting method that leverages preliminary LLM annotations to generate final annotation results (Additional file 1: Fig. S19B). The automated annotation results demonstrate strong concordance between the automated summary annotations and the original cell annotations comprising each cluster (Additional file 1: Fig. S19C).

Marker gene analysis revealed typical skin microenvironment cell types with expression profiles consistent with well-known markers (Fig. 5E). We then sought to confirm findings from previously published large-scale datasets. Initially, we isolated T cells and reconstructed the kNN-graph and UMAP (Fig. 5F). Consistent with earlier studies which revealed higher proportion of innate lymphoid cells (ILCs) in fetal skin, characterization of the ILC marker gene *KLRB1* confirmed its elevated expression in neonatal skin (Fig. 5G) [39]. Additionally, we identified distinct T cell expression patterns across disease states, including characteristic expression of *IL13* and *IL22* in AD, IL17 family genes in psoriasis, and *IFNG* in granuloma annulare (GA) (Fig. 5H). Concurrently, elevated *PTPRC* (encoding CD45) expression in immune-inflammatory lesion cells across all conditions indicated increased blood immune cell infiltration (Fig. 5H).

### Subcluster analysis identified distinct proliferating keratinocytes subtypes in psoriasis

Keratinocytes often exhibit pronounced phenotypes in autoimmune diseases, displaying high proliferation activity and impaired differentiation despite diverse inflammatory signals. To explore distinct keratinocyte states across diseases, we conducted a focused analysis on isolated keratinocyte populations.

**Fig. 6:**
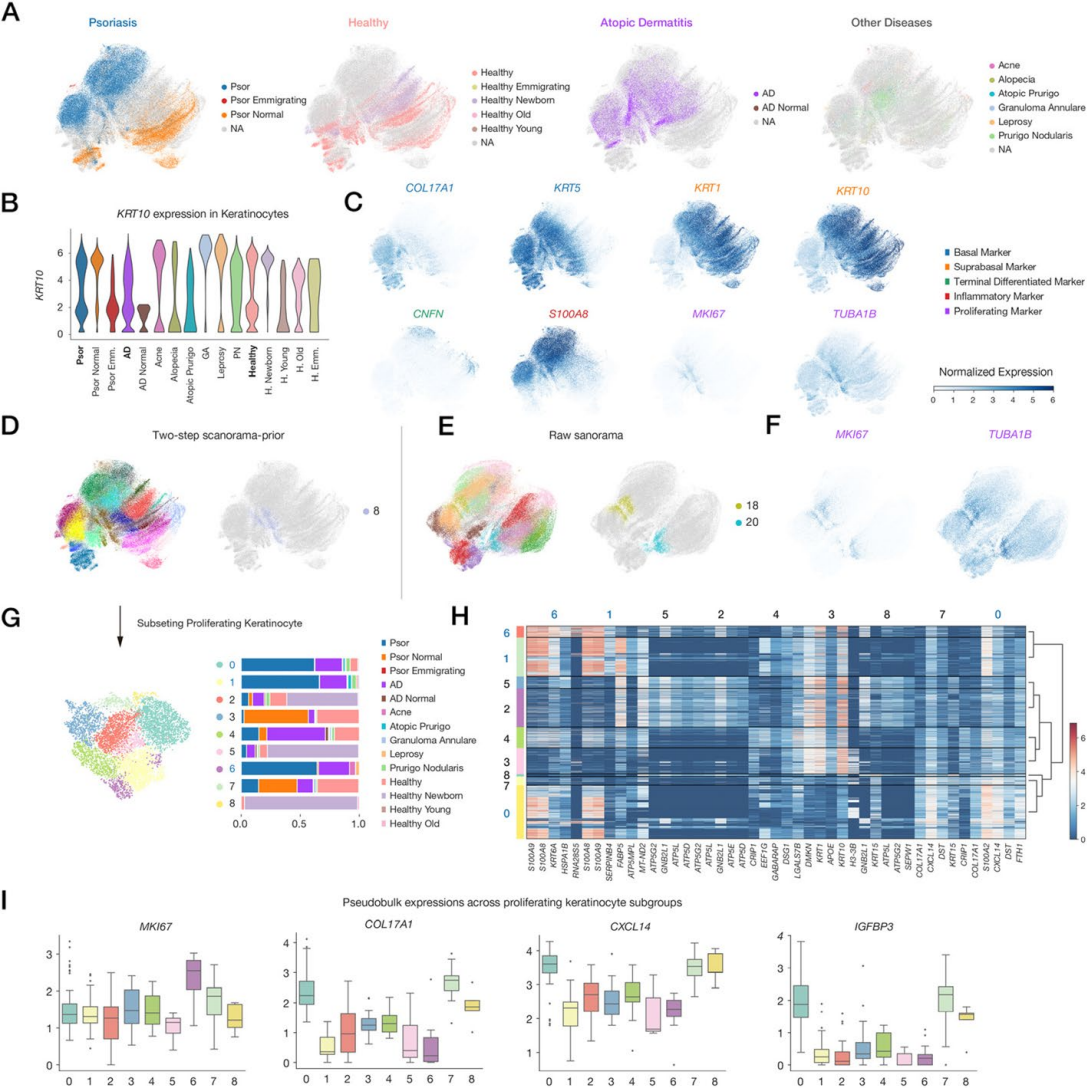
Analysis of keratinocyte subpopulations in integrated datasets. **A** UMAP visualization demonstrating differential keratinocyte differentiation trajectories across disease categories (from left to right): psoriasis, healthy controls, atopic dermatitis, and other skin disorders. **B** Violin plot showing *KRT10* expression levels as a keratinocyte differentiation marker across different phenotypes in keratinocyte subpopulations. **C** UMAP visualization of marker gene expression in keratinocyte subpopulations. **D** UMAP visualization of keratinocyte subclustering using scExtract’s two-step integration derived scanorama_prior embedding (left) and the identified high-proliferative keratinocyte subpopulation (right). **E** UMAP visualization of keratinocyte subclustering using original scanorama integration embedding (left) and the two distinct high-proliferative keratinocyte subpopulations identified (right). **F** UMAP visualization showing the expression of proliferation marker genes in the original scanorama integration embedding. **G** Analysis of high-proliferative keratinocyte subcluster composition across different annotated labels. **H** Heatmap plot showing marker genes expression across subclusters of proliferating keratinocytes. **I** Box plot showing pseudo-bulk expression levels of *MKI67*, *COL17A1*, *IGFBP3*, and *CXCL14* across distinct subclusters

UMAP visualization revealed multiple differentiation routes from progenitor to terminal keratinocytes (Fig. 6A). The psoriatic branch showed markedly elevated expression of inflammatory molecules such as *S100A8* (Fig. 6C), while keratinocytes from non-lesional psoriatic skin exhibited phenotypes similar to healthy keratinocytes, occupying a separate branch. Interestingly, AD keratinocytes were uniformly distributed between these two branches, predominantly occupying intermediate differentiation states (Fig. 6A). Analysis of *KRT10* expression provided further insights. Despite a higher proportion of *KRT10*-expressing cells within the AD population, overall expression levels were lower compared to other disease states (Fig. 6B). This pattern suggests a higher proportion of differentiated cells in AD, but with a lower degree of differentiation.

We evaluated the performance differences between prior-knowledge-enhanced integration methods and direct integration approaches in distinguishing fine-grained cell types (Methods). Our analysis revealed that two-step scanorama-prior effectively integrated cells across different batches, as exemplified by the successful clustering of proliferative keratinocytes from multiple batches (Fig. 6D). In contrast, the original integration method failed to properly merge these keratinocyte populations due to insufficient batch effect correction (Fig. 6E, F). Additionally, prior-knowledge-enhanced integration methods showed better preserved differentiation trajectories, whereas the original method tended to form more compact, spherical clusters.

We isolated proliferating keratinocyte subpopulations based on cell division-related marker genes like *MKI67* and performed subcluster analysis (Fig. 6C). Analysis of disease-specific proportions within subclusters revealed shared and disease-enriched populations (Fig. 6G). Clusters 2 and 7 were shared across diseases and healthy states, while psoriasis-enriched (clusters 0, 1, 6) and AD-enriched (clusters 4) populations were also identified.

Differential gene expression analysis of these subclusters revealed distinctive features (Fig. 6H). The predominant psoriasis-associated cluster (cluster 0) exhibited high *COL17A1* expression, potentially linked to selection pressures under proliferative signals [55]. Pseudo-bulk analysis revealed elevated *IGFBP3* expression in this cluster (Fig. 6I). Previous immunohistochemistry studies have localized *IGFBP3* expression to basal layer cells above epidermal papillae, a population characterized by lower proliferation capacity [56]. Furthermore, *CXCL14*, previously associated with anti-inflammatory effects in psoriasis, was also highly expressed in this cluster (Fig. 6I), in contrast to its overall decrease in psoriatic lesions [57]. Notably, a small cluster of cells from normal neonates exhibited similar characteristics. However, when comparing overall proliferative cell proportions, *CXCL14* ^+^ psoriatic keratinocytes increased from 35 to 58%, while the corresponding total proportion of healthy cells decreased from 27 to 13%. While typically associated with anti-inflammatory properties and lower proliferation activities, these cells appear to show altered gene expression patterns under psoriasis-related conditions. The elevated *COL17A1* expression might reflect changes in response to increased proliferative pressure of psoriatic lesions. However, further research is needed to fully elucidate the mechanisms underlying these gene expression changes and their functional implications in the context of psoriasis.

## Discussion

The power of single-cell RNA sequencing technologies in biological research has led to amazingly fast expansion of single-cell transcriptome data. However, many studies interpret datasets within their own constraints, limiting broader perspectives. Cross-dataset comparisons have emerged as a valuable tool for expanding our understanding, while flexible and adaptable reference datasets can yield novel insights in studies focused on specific diseases or biological processes. Nevertheless, in many articles, single-cell level annotations are not always uploaded to public repositories in a precise manner. Consequently, researchers attempting to reproduce these studies often engage in redundant work, with the workload increasing proportionally to the number of datasets incorporated. scExtract streamlines this process by leveraging large language models’ text information extraction capabilities to eliminate repetitive labor (Fig. 1B). This enables researchers to focus more intently on elucidating biological significance, rather than data preprocessing.

We propose that annotation and integration processes in constructing integrated datasets are mutually reinforcing. Previous attempts to utilize annotation information have been limited by annotation variability and inadequate standardized cell classification systems. This has restricted the application of methods like scANVI [58] and scGen [59], which rely on strict string matching, while approaches such as cellhint utilizes clustering information rather than cell type annotations. In cases where hard classification is challenging, continuous representation of annotations offers a promising alternative. While scExtract, employing current general-purpose text-to-embedding models, has shown corrective capabilities (Fig. 3A–D), these models lack domain-specific expertise. We anticipate the development of more specialized text-to-embedding models tailored to the biological domain, or even fine-tuned for cell type classification. Such advancements would enable more nuanced extraction of the biological significance from labels. We also envision the incorporation of continuous cell labels into deep learning methods like scANVI, potentially enhancing their adaptability to real-world datasets.

Despite demonstrating significantly higher accuracy compared to existing automatic annotation methods, the pre-harmonized results from scExtract should be interpreted with caution, particularly regarding the nomenclature of more refined cell subtypes. We recommend using cellhint or cellhint-prior to refine cell type annotations, leveraging other datasets as references. To facilitate user verification and potential manual correction of annotations, scExtract generates comprehensive log files. These logs contain the reasoning behind specific cell group annotations and the expression levels of queried genes across different clusters during the re-annotation process.

In this study, we utilized the scExtract pipeline to automatically integrate 14 datasets, constructing a comprehensive skin dataset spanning multiple diseases and physiological states (Fig. 5A). Our analysis initially validated conclusions from previous datasets, then focused on keratinocytes, particularly proliferating keratinocytes, which were isolated and independently analyzed. Our investigation identified distinct subpopulation expansion specific to psoriasis, one of the most prevalent autoimmune skin diseases (F ig. 6I), which is associated with anti-inflammatory functions. While past research on autoimmune diseases has predominantly centered on immune cells, our study highlights the importance of keratinocytes, which constitute the majority of cells in the skin microenvironment but have been understudied. This example underscores the need for more detailed and standardized research into keratinocyte subpopulations, which could enhance our understanding of their specific roles under various conditions. Furthermore, our application of scExtract demonstrates its versatility and potential to significantly reduce the manual workload associated with meta-analyses of subpopulations [60]. Additionally, scExtract can assist researchers in constructing customized reference datasets tailored to their specific areas of interest.

In conclusion, our study not only provides valuable insights into the cellular dynamics of skin autoimmune diseases but also showcases the power of automated data integration and analysis tools like scExtract. As we continue to unravel the complexities of cellular heterogeneities in health and disease situations, such tools will be instrumental in accelerating discoveries and fostering more comprehensive understanding of biological systems.

## Conclusions

This study is a novel comprehensive framework for fully automated single-cell RNA-seq data annotation and integration using large language models. scExtract addresses the critical bottleneck of processing abundant public datasets by automatically extracting experimental parameters and biological knowledge from research articles. Our annotation approach, incorporating article-derived prior knowledge and self-reflective capabilities, consistently outperformed established reference transfer methods across diverse tissue types and cell populations. The integration pipeline, featuring scanorama-prior and cellhint-prior, demonstrates superior batch correction while preserving biological diversities compared to conventional methods that ignore annotation information. This framework is particularly valuable for discovering rare cell types and novel biological insights that might be overlooked by reference-based approaches. We successfully applied scExtract to construct a comprehensive human skin atlas comprising 440,000 cells from 14 datasets, revealing disease-specific keratinocyte subpopulations and inflammatory signatures across multiple autoimmune conditions. The identification of CXCL14 ^+^ proliferating keratinocytes in psoriasis exemplifies scExtract’s capability to uncover previously understudied cellular dynamics in complex diseases. By eliminating labor-intensive manual preprocessing and enabling cost-effective integration of published datasets, scExtract democratizes large-scale single-cell analysis and accelerates the construction of tissue-specific atlases. Our framework provides essential infrastructure for the single-cell omics community to harness the wealth of existing data, facilitating reproducible research and advancing our understanding of cellular heterogeneities in health and disease.

## Methods

### scExtract process details

scExtract requires two primary inputs: the article in PDF format and corresponding raw count-level dataset in h5ad format. To facilitate potential sample-level batch correction, a Batch key must be specified in the “obs” field, typically using the filename.

### Processing pipeline

Like manual processing workflows, scExtract’s pipeline consists of four main steps: filtering, preprocessing, clustering, and two rounds of annotation. The specific prompts for each step are available in the source code.

(1) Filtering.

This stage eliminates cells with poor quality control (QC) metrics. Thresholds are applied to the number of genes expressed per cell, total counts per cell, and the number of cells expressing each gene. Default parameters, such as a minimum of 300 genes expressed per cell, are preset for common 10X single-cell datasets. Additional filters, including upper limits on genes per cell and proportions of mitochondrial and ribosomal genes, are only applied if explicitly mentioned in the article and parsed by the language model.

(2) Preprocessing.

This stage prepares the data for clustering. While most parameters use default settings, key adjustable parameters include the number of highly variable genes selected, the neighborhood size for kNN graph construction, and the option for sample-level batch correction using harmonypy [61].

(3) Clustering.

Clustering methods (Leiden or Louvain) and the estimated number of cell clusters are determined at this stage. scExtract selects an appropriate resolution parameter based on the inferred cluster number. In this step, more sophisticated models generally yield more accurate parameter estimates.

(4) Annotation.

To further optimize costs, we cleared the previous question-answering cache context before the annotation phase and utilized separate tool models for specific tasks where extensive context was unnecessary. Initial annotation uses the top 10 highly variable genes for each cluster, identified through Wilcoxon rank-sum tests. This strategy has been shown to yield optimal performance [7]. scExtract integrates this information with background knowledge to annotate cell clusters. Annotations include cell type, confidence level, sample tissue origin, disease state, and developmental stage. It should be noted that these annotations carry inherent variability due to LLM outputs and should be interpreted with appropriate caution.

(5) Re-annotation (optional).

In this step, scExtract allows the language model to select genes for querying, potentially improving annotations. As language models are not sensitive to numerical inferences, cluster-level expression data is first summarized into natural language by a tool model before being input into scExtract for judgment and re-annotation.

### Output and documentation

In addition to the processed dataset, scExtract generates a config file documenting all processing parameters and a log file detailing the reasoning behind parameter choices and annotations at each step (Fig. 1B). For multi-dataset annotations, cross-dataset correction is recommended. For single dataset annotations, users should carefully review the log file contents.

### Cellxgene benchmark dataset preparation

To evaluate scExtract’s performance, we utilized manually processed datasets from cellxgene. Eighteen datasets were selected (Additional file 2: Table S1), balancing random selection with sample diversity. We prioritized datasets containing fewer than 50,000 cells per individual set, as larger datasets often comprise multiple sub-datasets with more chance to be uploaded to public cohorts, diminishing the need for automatic annotation. We downloaded the original matrix data uploaded by study authors and aligned them with cellxgene’s processed data using cell barcodes. During this process, two datasets were excluded due to lack of original author-defined cell type column, leaving six datasets for benchmarking.

### Automatic annotation with established methods

For singleR, we used python implemented SingleR with “HumanPrimaryCellAtlas” as the reference dataset for label transfer annotation. This choice prioritizes method generalizability over optimizing SingleR’s performance with specialized datasets, aligning with our focus on automated processing. We employed CellTypist v1.6.3 [30] for our analysis. As CellTypist does not provide cross-tissue reference data, we utilized LLMs to automatically extract tissue types from datasets and performed label transfer using corresponding reference data. Moreover, it is important to note that the fundamental limitation in label transfer algorithm, the inability to identify and annotate novel cell types, persists regardless of the reference dataset used. We applied the same approach to scType; however, due to scType’s limited tissue type coverage, it successfully annotated fewer samples compared to the aforementioned methods.

### Benchmark cellxgene dataset annotation accuracy

Consistent with our use of LLMs, we adopted a text-to-embedding approach, with configurable models specified in the scExtract config file. Ontology-based method is also supported in scExtract.benchmark function setting method parameter to “ols_api”. However, this method is constrained by naming style limitations in its initial string-matching step from custom annotation to cell ontology nodes trough Ontology Lookup Service. While the original version [7] used a binary scoring system (1 for exact matches, 0.5 for shared parent classes), we implemented a more nuanced approach. We calculate the Jaccard coefficient using all hierarchical nodes in the cell classification tree, resulting in a more universally applicable and granular evaluation. The primary challenge with the text-to-embedding method lies in its training on general models, where cell type semantics may be influenced by other linguistic contexts (Additional file 1: Fig. S2D). We anticipate that a future fine-tuned model could better differentiate the biological significance of cell types, potentially improving the accuracy of automated annotations.

### Prompt variation designs

To explore how different prompts influence task performance, we developed five variants of our original prompt, including Instruction_Reordering, which simply rearranged the narrative sequence; Terminology_Modification, which replaced terms with synonyms; Enhanced_Guidance, which incorporated more detailed descriptions and instructions; Minimalistic_Annotation, which used the minimal possible instructional content; and Structural_Reformatting, which employed JSON format for output, while other prompts utilized YAML-like format. The contents of prompts variants can be inspected at the github repository for reproduction (https://github.com/yxwucq/scExtract_reproduce/blob/master/scripts_revision/prompt_variation.py). We evaluated the impact of different prompts on both preprocessing and annotation stages separately, creating five variants each for the filter parameter extraction and annotation prompts, and repeated the evaluations for three times on Sample 11 to assess the performance of different prompt configurations.

### Scanorama-prior integration methods with prior knowledge

We utilize cell type similarity matrices as previously mentioned to introduce prior information on cell cluster similarities. In the mutual nearest neighbor (MNN) construction process, cell distance matrices are weighted by naming similarities:

$${{\text{d}}{\prime}}_{ij}={\text{d}}_{ij}/{\text{M}}_{IJ}$$

where ${{\text{d}}{\prime}}_{ij}$, ${\text{d}}_{ij}$, and ${\text{M}}_{IJ}$ are modified distance between cell *i* and cell *j*, raw distance between cell *i* and cell *j*, cell type similarity between cell type *I* and cell type *J*. The general-used text to embedding model’s cell naming distances are typically more than 0.3, allowing for meaningful differentiation without introducing extreme outliers. In faster kNN graph construction methods using annoy index, scanorama-prior first expands the candidate cell pool to five times (configurable) the size of *k*. It then performs weighted distance calculations within this expanded candidate list to select the *k* nearest neighbors.

During the distance reduction process, cells lacking adjacency relationships with the target dataset’s cells are shifted closer to expression-similar cells within the same group, minimizing interference from other group displacement vectors. Additionally, a cluster center-based displacement vector is applied based on the annotation similarity between cells and their corresponding target dataset cells. This is represented as:

$${{\text{Bias}}{\prime}}_{ij}={\text{Bias}}_{ij}+{\text{M}}_{IJ}\times ({v}_{i}-{v}_{I}-{v}_{j}+{v}_{J})$$

where ${\text{Bias}}_{ij}$, ${\text{M}}_{IJ}$, ${v}_{i}$, ${v}_{I}$, ${v}_{j}$, ${v}_{J}$ are original bias between cell *i* and *j*, cell type similarity between cell type *I* and cell type *J*, embedding vector of cell *i*, mean embedding vector of cell type *I*, embedding vector of target cell *j*, embedding vector of target cell type *J*. Consequently, higher annotation consistency results in new embeddings being shifted to maintain relative positions within the new cluster, enhancing integration.

### Cellhint-prior harmonization methods with prior knowledge

We utilize cell type similarity matrices to incorporate prior information on cell cluster similarities. The integration process begins by calculating the raw distance matrix between cells and cell types. The raw distance matrix is then normalized by dividing it by the mean distance across all cell pairs and converted into a similarity matrix using negated exponential transformation. When embedding dictionaries are provided, a prior similarity matrix is computed using cell type embeddings. The raw distance matrix is then normalized to a similarity matrix:

$${{\text{S}}{\prime}}_{iJ}=\upbeta {[(1-{{\alpha }}_{i})\text{S}}_{iJ}+{{{\alpha}}_{i}\text{M}}_{IJ}]+\left(1-\upbeta \right){\text{S}}_{iJ}$$

where ${{\text{S}}{\prime}}_{ij}$, $\upbeta$, ${{\alpha }}_{i}$,${\text{S}}_{ij}$, and ${\text{M}}_{IJ}$ are adjusted similarity of cell *i* and target cell type *J*, hyper parameter for the effect of prior information (default set to 0.1), inversion of clarity score which is calculated based on the entropy of cell *i* to all cell types, raw similarity of cell *i* and target cell type *J*, and cell type similarity between cell type *I* and cell type *J*. The clarity score, which quantifies the distinctiveness of a cell’s expression profile, serves as a modulator in this process. When the clarity score decreases, indicating difficulty in distinguishing cell types based solely on expression data, the influence of prior knowledge becomes more pronounced. Conversely, for cells with high clarity scores, the algorithm relies more heavily on the expression-based similarities.

### Pancreas dataset benchmark

For the pancreas dataset benchmark, we evaluated scanorama and scanorama_prior using identical parameters. Log1p-transformed data with highly variable genes were used as model inputs. Integration was performed using the integrate_scanpy function of both methods, with approx = False and knn = 30. For evaluation, neighborhood graphs were generated for both embeddings using n_neighbors = 30, and UMAP embeddings were created with default parameters. All embeddings were evaluated using single-cell benchmarking metrics provided by scib_metrics [6]. For cellhint and cellhint_prior, we applied consistent default parameters as recommended by the original methods [9], using 50-dimensional PCA embeddings as input without the PCT method. Cell type embeddings were generated using OpenAI’s text-embedding-3-large model accessed through Azure API.

### Label change experiment

For label change experiments, we manually modified specific cell types in selected datasets and evaluated using the aforementioned integration methods. We conducted five batch experiments, each incorporating three types of modifications described in the paper: Fuzzy match, Unbiased error, and Biased error. Integration methods remained consistent with above. To minimize background interference, we isolated cells with modified labels for subsequent evaluation. Integration metrics were calculated using ilhouette_score, adjusted_rand_score, and normalized_mutual_info_score function from sklearn.metrics module. Additionally, we performed unsupervised clustering using Leiden algorithm (resolution = 0.7) and applied major voting to the modified cell types to generate consensus cell type annotations. The results were analyzed using confusion matrices.

### Time consumption across diverse methods

We conducted a comprehensive evaluation of runtime performance on both a PC and a GPU-accelerated server. The PC was equipped with an Intel Core i5-13600 K processor, while the server used Intel(R) Xeon(R) Gold 6132 CPU @ 2.60 GHz with an Nvidia Tesla V100-PCIE-32 GB GPU. To generate a large-scale dataset, we utilized the pancreas dataset and employed a replication-based approach. Specifically, the original dataset was replicated three times, with each copy assigned a unique index to serve as the initial input for concatenation. This input was then progressively concatenated to simulate data scales ranging from 1 × to approximately 20x (equivalent to 1 million cells).

Each method was systematically integrated using the aforementioned approach, with the focus solely on the time required for the integration process. Specifically, we benchmarked the runtime of cellhint.harmonize, scanorama.integrate_scanpy, and their respective prior-incorporated variants. On the GPU-enabled server, we activated GPU acceleration by setting use_gpu = True, leveraging CuPy for enhanced computational efficiency. To mitigate the risk of memory overflow, we configured the batch_size parameter for the scanorama-related methods to 1000 cells.

### Large-scale dataset benchmark

The integration and benchmarking methodologies employed were consistent with those previously described. Notably, within the Human Oral Cell Atlas dataset, we excluded a dataset labeled as “multiple” due to the inability to accurately assess its independence. No modifications were made to the remaining datasets.

### Automatic integration of 14 skin datasets

We initially selected 20 datasets for our integration study. One dataset was excluded due to its murine origin, and another for being a duplicate. Two datasets were omitted owing to missing matrix files. A dataset with atlas-level cell numbers was reserved for subsequent assessment of result reproducibility. Another dataset was excluded due to insufficient cell count. This curation process yielded 14 datasets suitable for integration. To maintain data diversity while managing computational load, datasets exceeding 60,000 cells were randomly downsampled to this threshold.

The integration process followed a standardized three-step protocol: initial automatic annotation using scExtract, followed by cell type harmonization via cellhint-prior, and finished in embedding integration using scanorama-prior. For the integrated dataset, we applied X_scanorama_prior with the parameter n_neighbors = 30 to compute the neighbors’ graph. Subsequently, we employed the Leiden clustering algorithm with resolution = 0.5 to partition the cells into distinct clusters. To further refine the annotation, we utilized the major_vote_top_clusters function provided by scExtract, which performs a consensus-based voting mechanism across individual dataset annotations to assign consistent cell type labels.

### Subcluster analysis and pseudo-bulk generation

For the subpopulation analysis of T cells and keratinocytes, we extracted the corresponding subsets from the large-scale integrated dataset. Given the reduced number of cells in these subsets, we adjusted the parameter n_neighbors to 15 to optimize the analysis. Using the top 30 principal components (PCs), we reconstructed the neighborhood graph and UMAP embeddings with both X_scanorama_prior and X_scanorama.

To mitigate the impact of batch differences between single cells on differential gene expression, we constructed pseudo-bulk samples using decoupleR v1.6.0 [62]. This approach helps to reduce the influence of technical variations between batches and provides a more robust foundation for identifying biologically relevant gene expression differences.

## Supplementary Information

Additional file 1. Contains all supplementary figures from S1 to S19.

Additional file 2. Table S1: Cellxgene datasets used for annotation accuracy evaluation.

Additional file 3. Table S2: Prompt variation experiments for sensitivity testing.

Additional file 4. Table S3: Skin scRNA-seq datasets selected for atlas construction.

## Data Availability

All datasets this paper analyzed are publicly available data. Accession numbers for used datasets are listed in Additional file 2: Table S1. Processed input dataset samples in the benchmarking section are available at 10.5281/zenodo.13827072 [63]. All original code of scExtract has been deposited on GitHub and is publicly available under https://github.com/yxwucq/scExtract [64] and open-sourced under BSD 2-Clause License. Modified version of integration methods are under https://github.com/yxwucq/cellhint_prior and https://github.com/yxwucq/scanorama_prior. All processing results and generated figures are available at https://github.com/yxwucq/scExtract_reproduce. They are also uploaded to Zenodo in a complete package under https://zenodo.org/records/15555221 [65].
