# Supplementary material for: scExtract: leveraging large language models for fully automated single-cell RNA-seq data annotation and prior-informed multi-dataset integration
Source: Genome Biol. 2025 Jun 19;26:174. doi: 10.1186/s13059-025-03639-x (PMC12178070; doi:10.1186/s13059-025-03639-x)
Supplement: Supplementary file 1 — Additional file 1. Contains all supplementary figures from S1 to S19. [file 13059_2025_3639_MOESM1_ESM.docx]

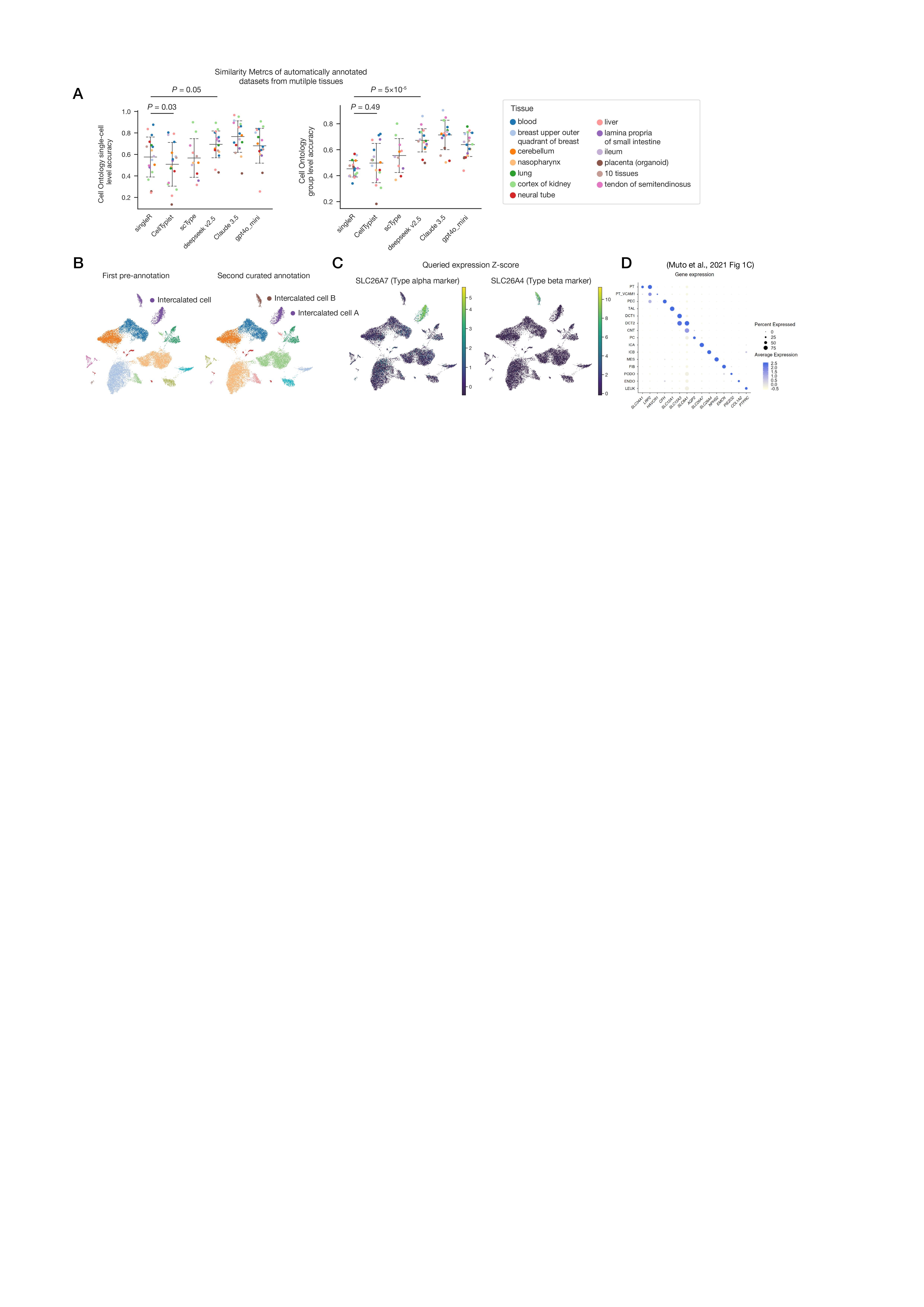


**Fig S1. Analysis of scExtract accuracy on cellxgene datasets**

(A) Dot plot illustrating the comparison of cell annotation similarity metrics on benchmark datasets, using gene ontology level similarity. The left side shows accuracy averaged at the single-cell level, while the right side shows accuracy averaged at the group level. p-values calculated using Wilcoxon signed-rank test.

(B) UMAP plot showing changed label after second-round curated annotation

(C) UMAP visualization demonstrating marker gene expression patterns distinguishing type α and β intercalated cells.

(D) Expression profiles of original marker genes in the human kidney dataset.


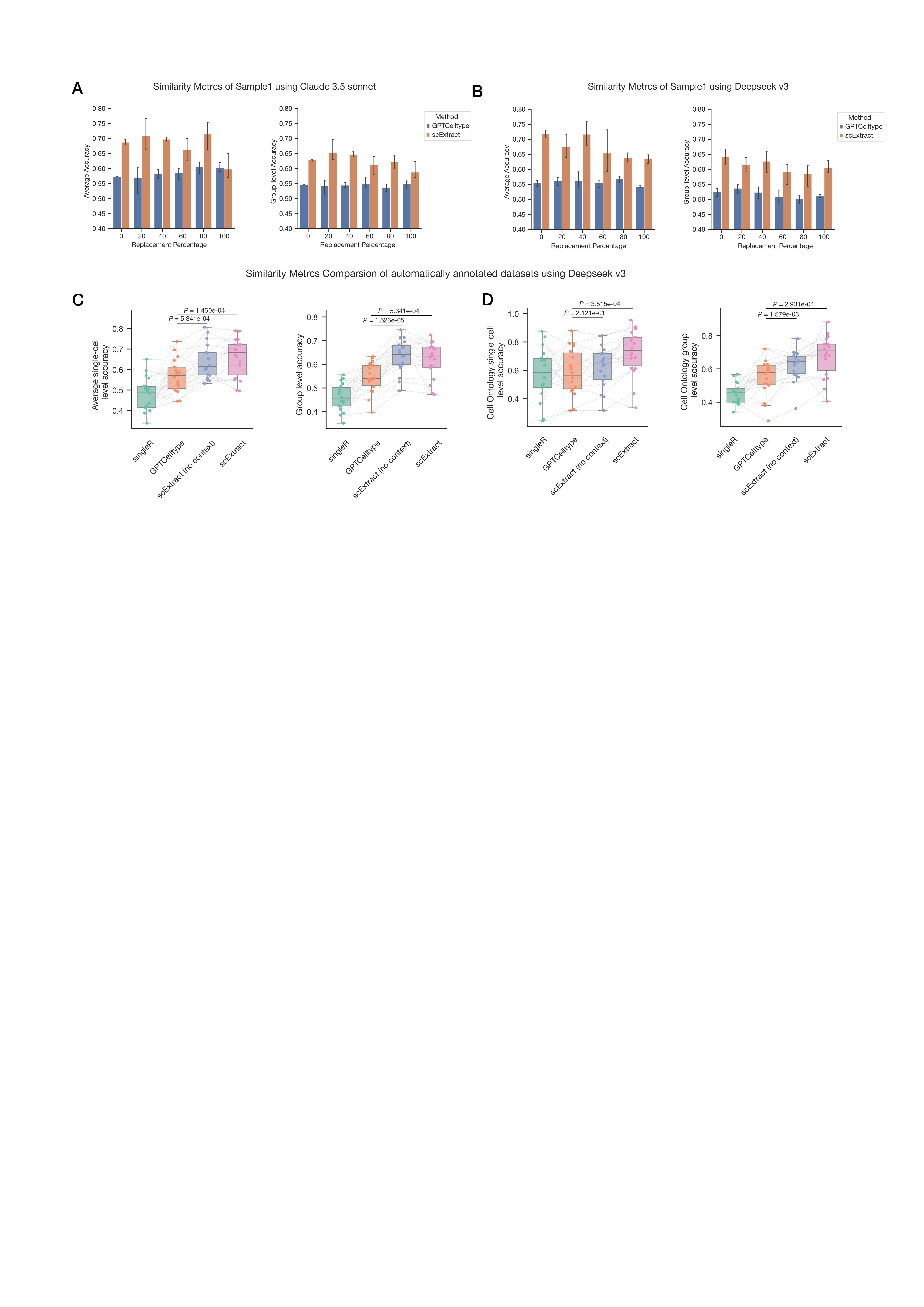


**Fig S2. Impact of confounding information on annotation accuracy and comparative performance with GPTCelltype**

(A) Bar charts describing the changes in accuracy when using Claude 3.5 sonnet for annotation. left panel: average cell annotation accuracy and right panel: group-level accuracy, as text information is progressively replaced with confounding content.

(B) Bar charts describing the changes in accuracy when using Deepseek v3 for annotation. left panel: average cell annotation accuracy and right panel: group-level accuracy, as text information is progressively replaced with confounding content.

(C-D) Dot plot illustrating the comparison of cell annotation similarity metrics across four different annotation methods on benchmark datasets with Deepseek v3 as the annotation model. (C.) shows text-to-embedding similarity measurements, while (D.) presents cell-ontology similarity metrics. Within each panel, the left side displays cell-level average accuracy, while the right side shows cluster-level accuracy. p-values calculated using Wilcoxon signed-rank test.


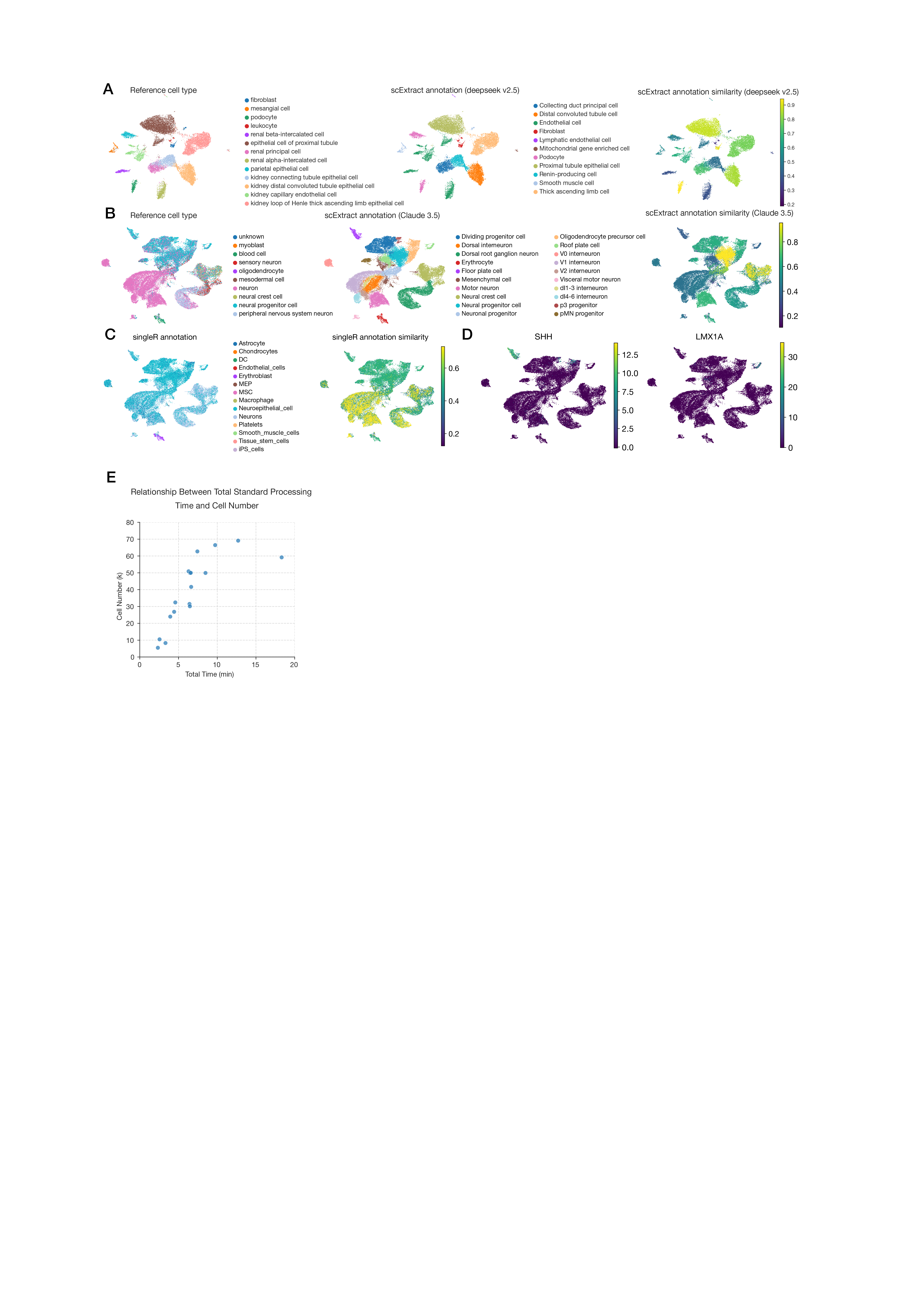


**Fig S3. scExtract accurately annotates single-cell RNA-seq datasets across diverse tissues**

(A) UMAP visualization of human kidney dataset cell type annotations generated by scExtract using Deepseek v2.5. Panels from left to right show curated cell type annotations, annotations generated by scExtract using Deepseek v2.5, and the text-to-embedding level accuracy of Deepseek v2.5 annotations.

(B) UMAP visualization comparing reference cell types (left) with scExtract annotations using Claude 3.5 (right) in the neurodevelopment dataset. Panels from left to right show curated cell type annotations, annotations generated by scExtract using Claude 3.5, and the text-to-embedding level accuracy of Claude 3.5 annotations.

(C) UMAP visualization comparing reference cell types (left) with SingleR annotations (right) in the neurodevelopment dataset. Panels from left to right show annotations generated by scExtract using SingleR and the text-to-embedding level accuracy of SingleR annotations.

(D) UMAP visualization showing expression levels of *SHH* (floor plate cell marker) and *LMX1A* (roof plate cell marker).

(E) Scatter plot showing how the time (minutes) required for scExtract's standard data processing pipeline changes as the number of cells (thousands) in the dataset increases. The processing time does not include dataset-specific steps, such as converting gene Ensembl IDs to gene names, which may be required for certain datasets.


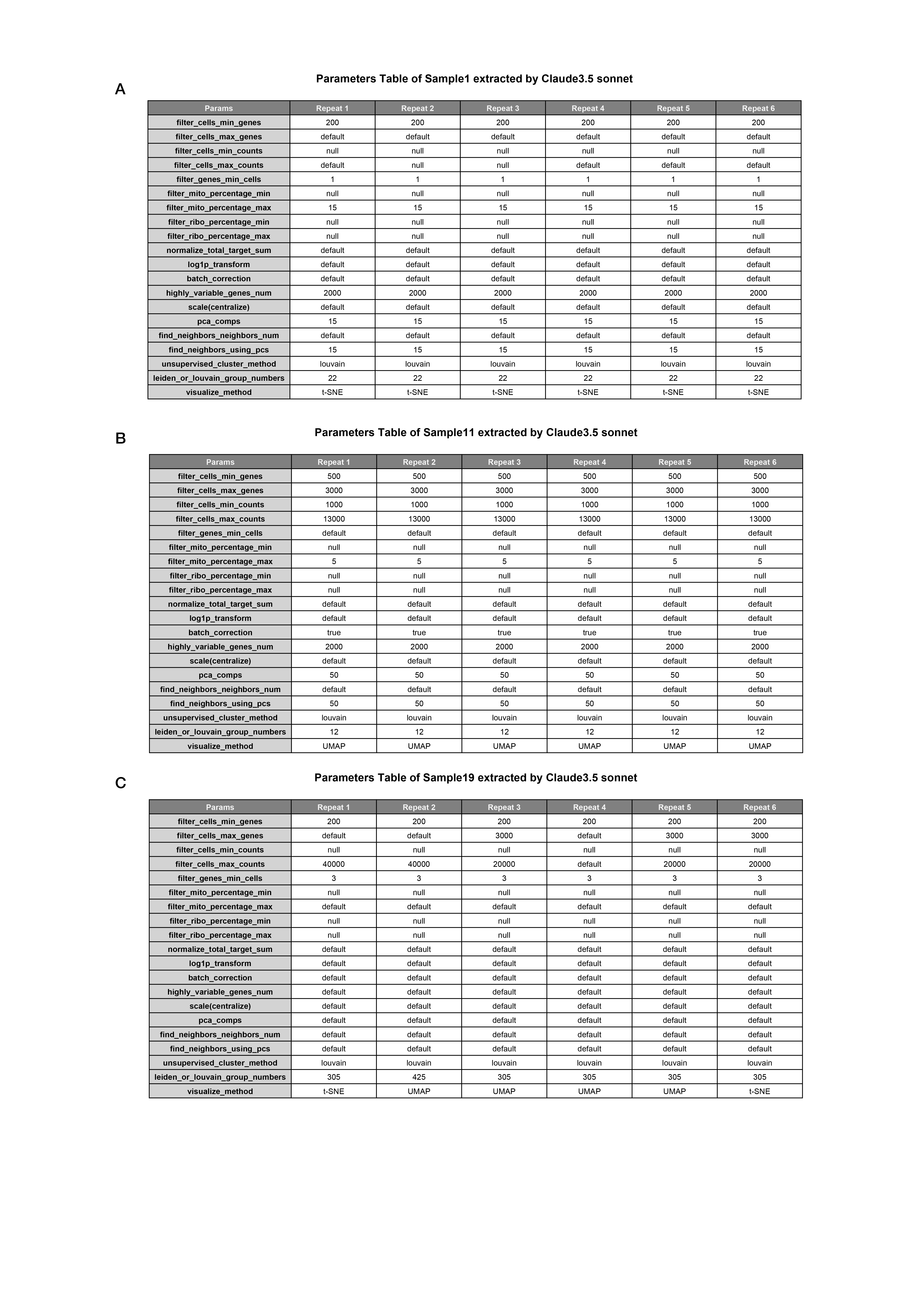


**Fig S4. Large language models can robustly extract most article preprocessing parameters**

(A-C) We processed three samples with six replicates using Claude 3.5, resulting in processing parameter tables for (A) Sample1, (B) Sample11, and (C) Sample19. Each row represents a preprocessing parameter, and each column represents a replicate. Most parameters remain stable across different replicates. For Sample 19, a preprint from medRxiv, scExtract's parameter extraction exhibited comparatively lower consistency. For optimal implementation, we specifically recommend: 1. Avoiding processing papers containing multiple datasets with fundamentally different content; 2. Avoiding pre peer-reviewed manuscripts, as they typically have not undergone content condensation, and extensive contextual information or potential errors may compromise the LLM agent's judgment.


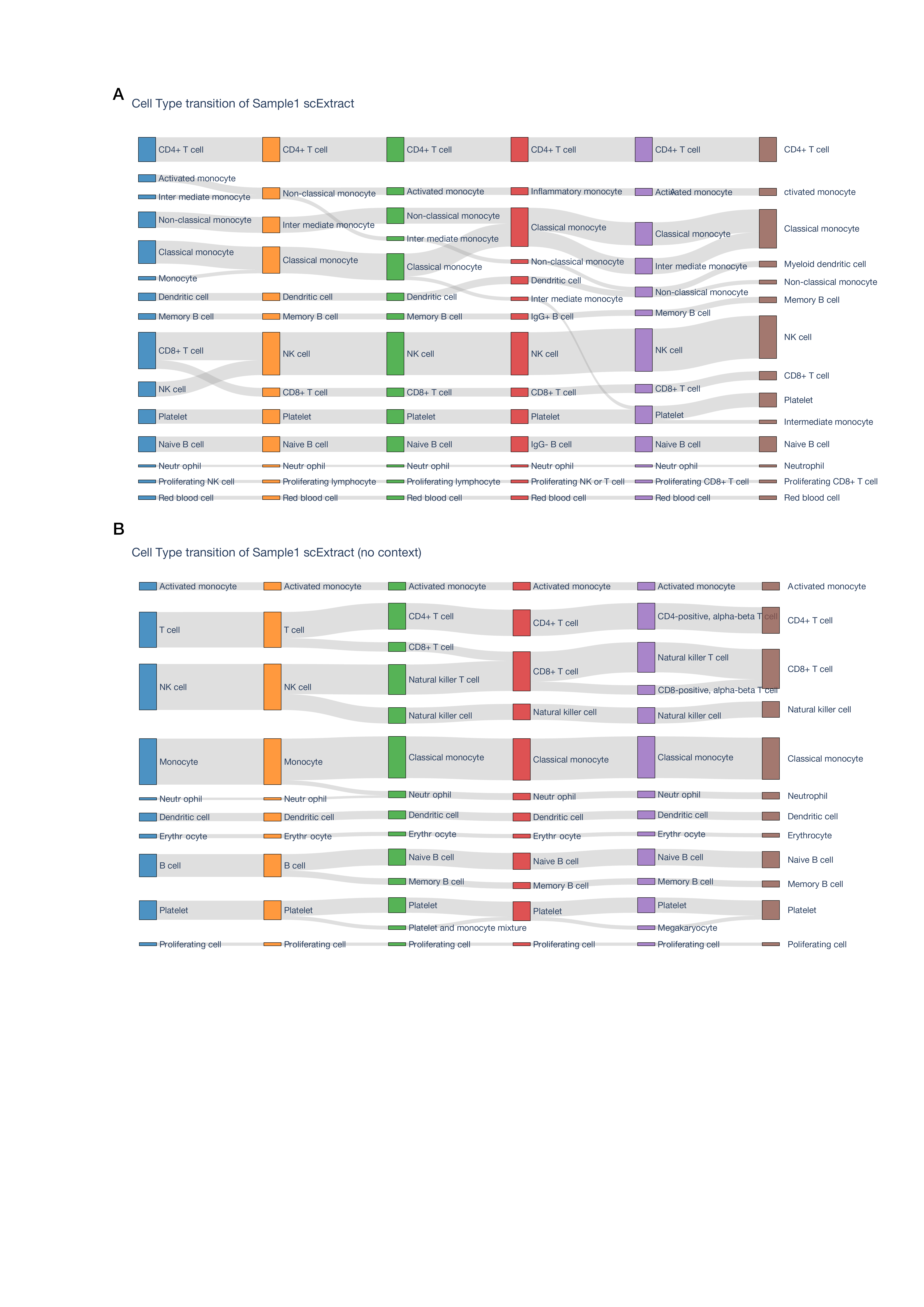


**Fig S5. Analysis of scExtract cell type annotation variation across different replicates in blood samples**

(A-B). Transition plot illustrating the changes in cell type annotations produced by (A) scExtract (B) scExtract (no context) across different replicates on sample1, with each column representing a replicate, for a total of 6 replicates.


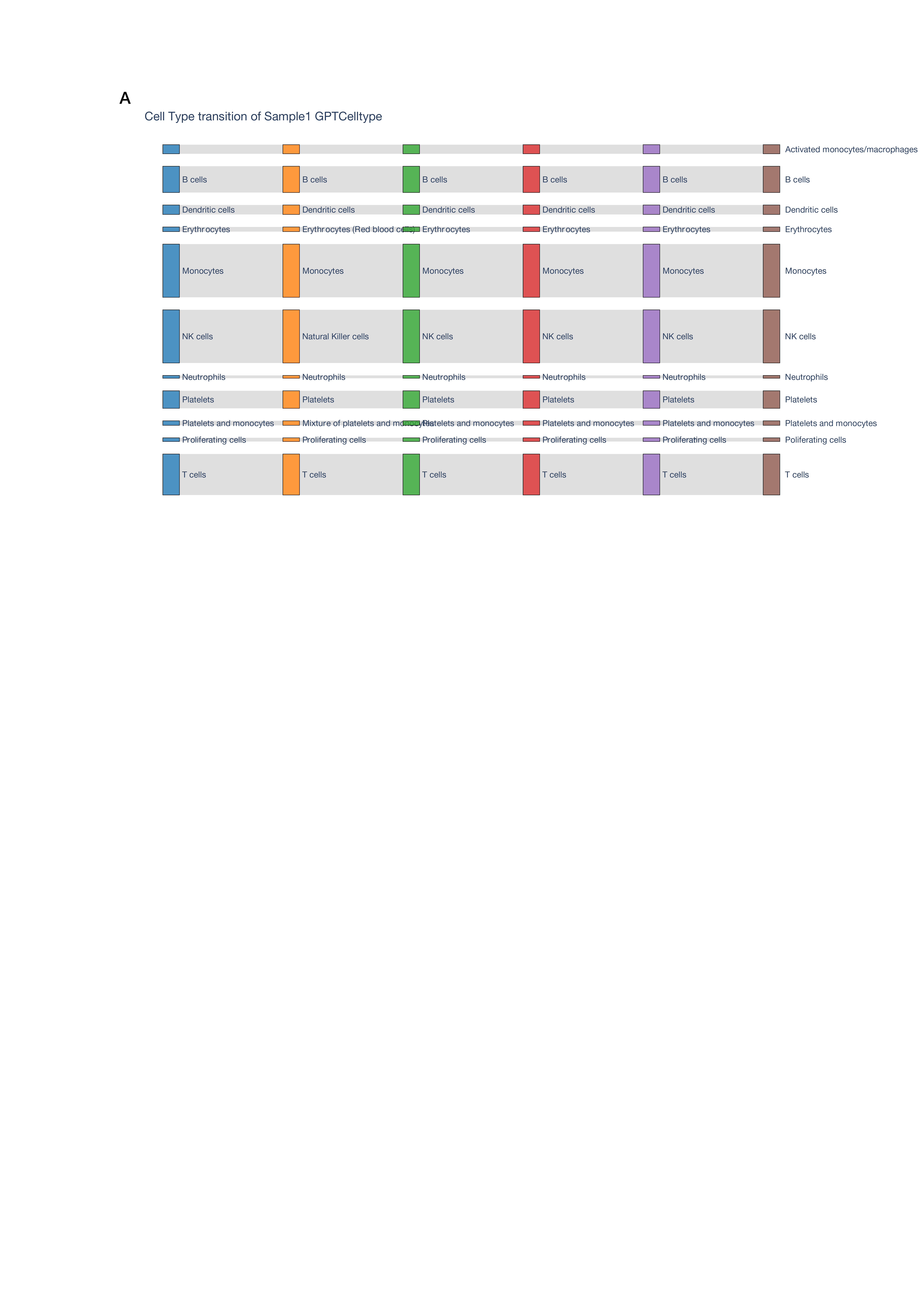


**Fig S6. Analysis of GPTCelltype annotation variation across different replicates in blood samples**

(A) Transition plot of GPTCelltype’s annotation on sample1, each column represents a replicate, with total amount of 6 replicates. To avoid legend overlap, lengthy annotations that were identical to those in the right-side legend have been erased from the figure. The erased annotations are consistent with those shown in the rightmost section.


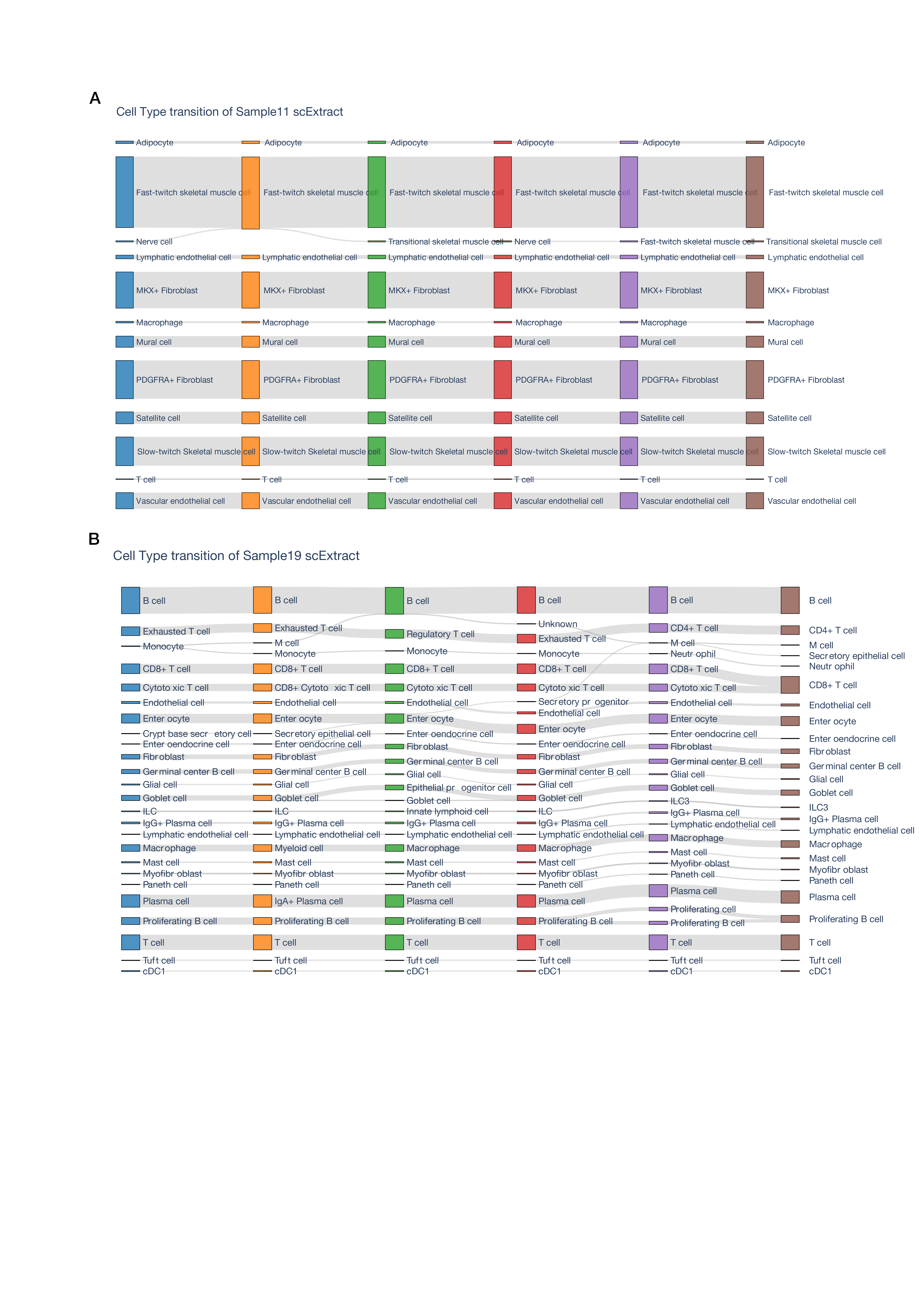


**Fig S7. Analysis of scExtract cell type annotation variation across different replicates in solid tissue samples**

(A-B). Transition plot illustrating the changes in cell type annotations produced by scExtract across different replicates on (A) sample11 (B) sample19, with each column representing a replicate, for a total of 6 replicates.


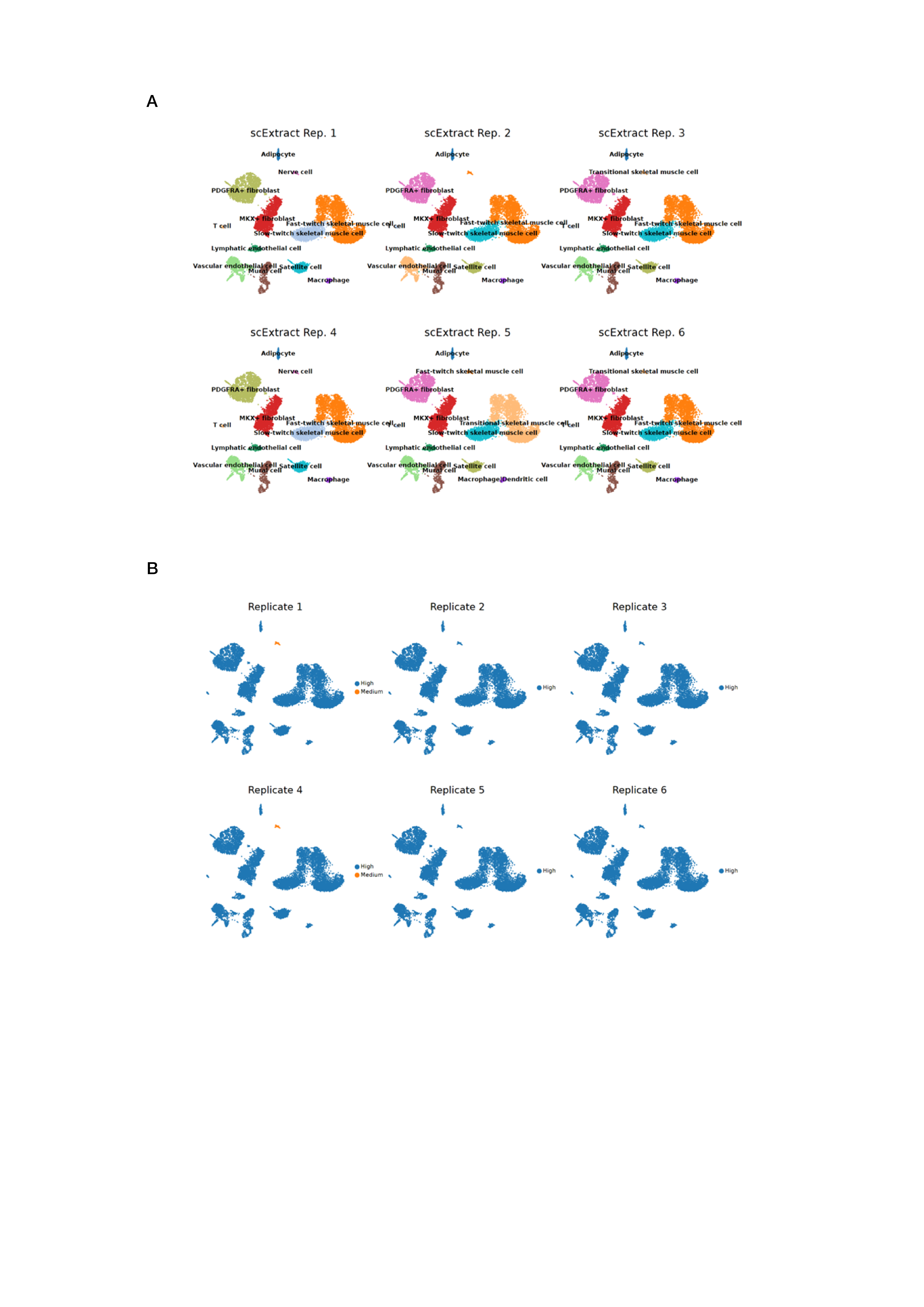


**Fig S8. Replication analysis of scExtract annotation performance and self-reported certainty**

(A) UMAP plot showing scExtract's annotation of sample11 dataset, each panel represents a replicate.

(B) UMAP plot showing scExtract's self-reported annotation certainty of sample11 dataset, each panel represents a replicate.


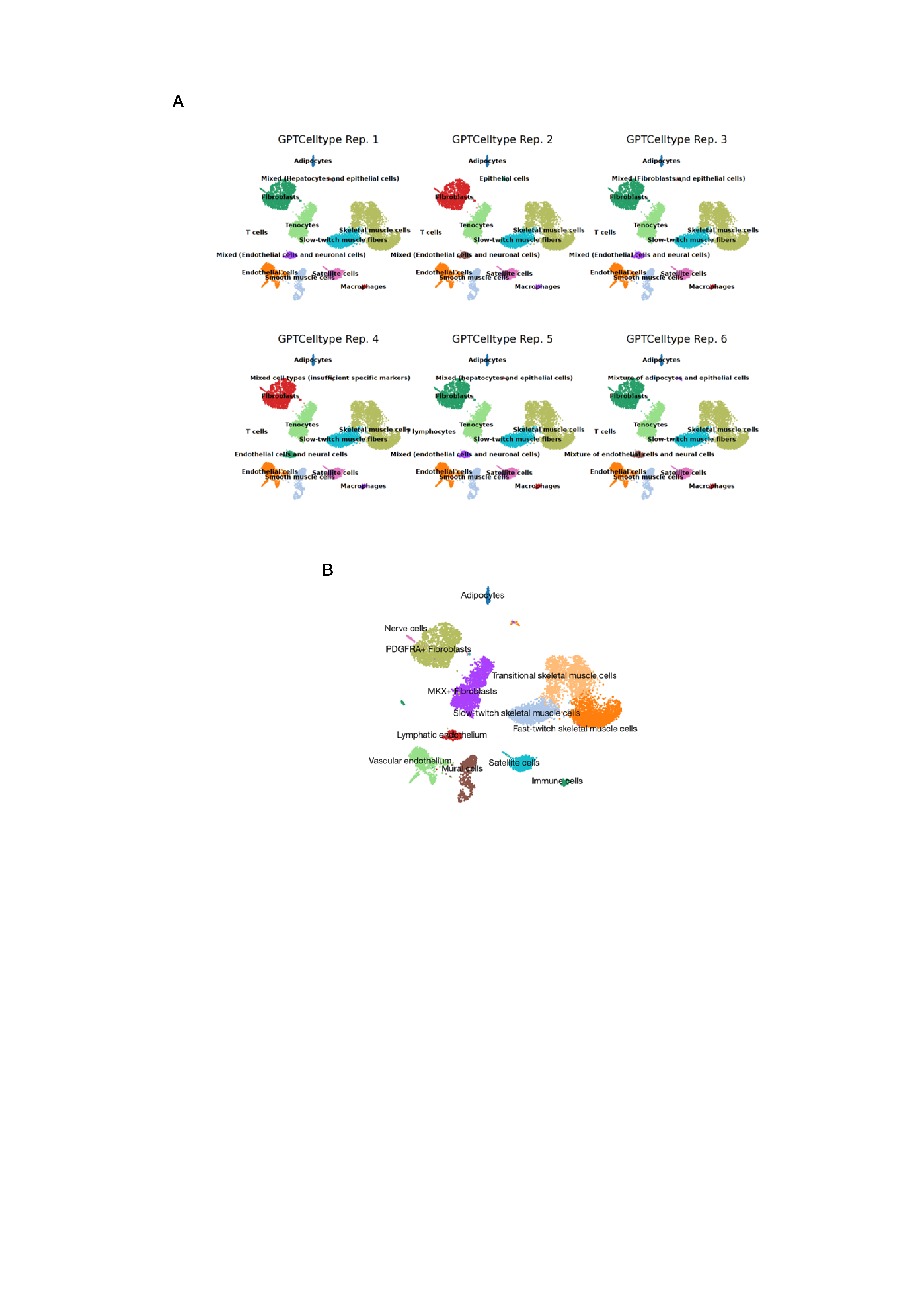


**Fig S9. Replication analysis of GPTCelltype annotation consistency and original curated annotations**

(A) UMAP plot showing GPTCelltype's annotation of sample11 dataset, each panel represents a replicate.

(B) UMAP plot showing original authors’ curated annotations of sample11 dataset

**A**


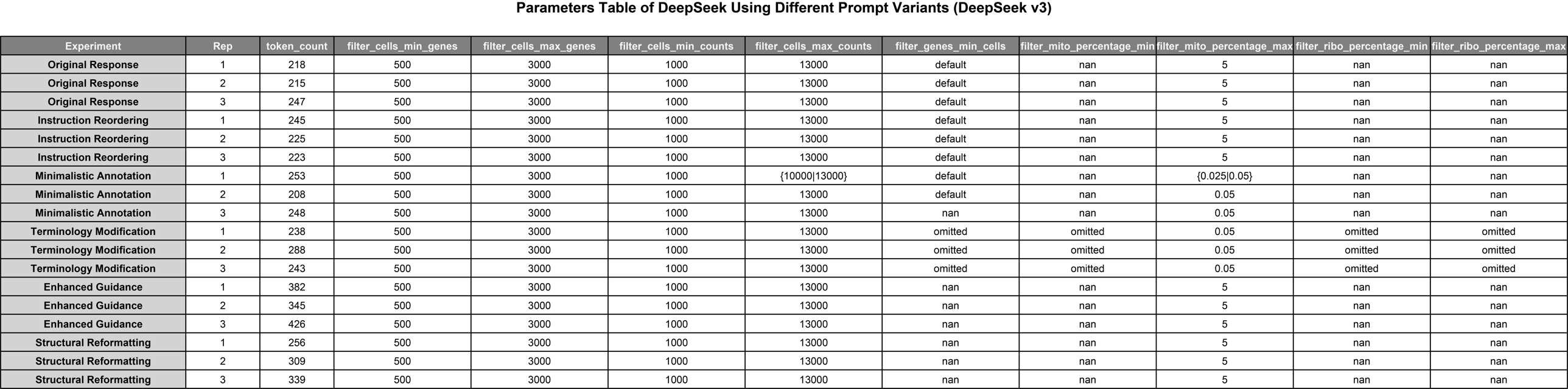


**B**


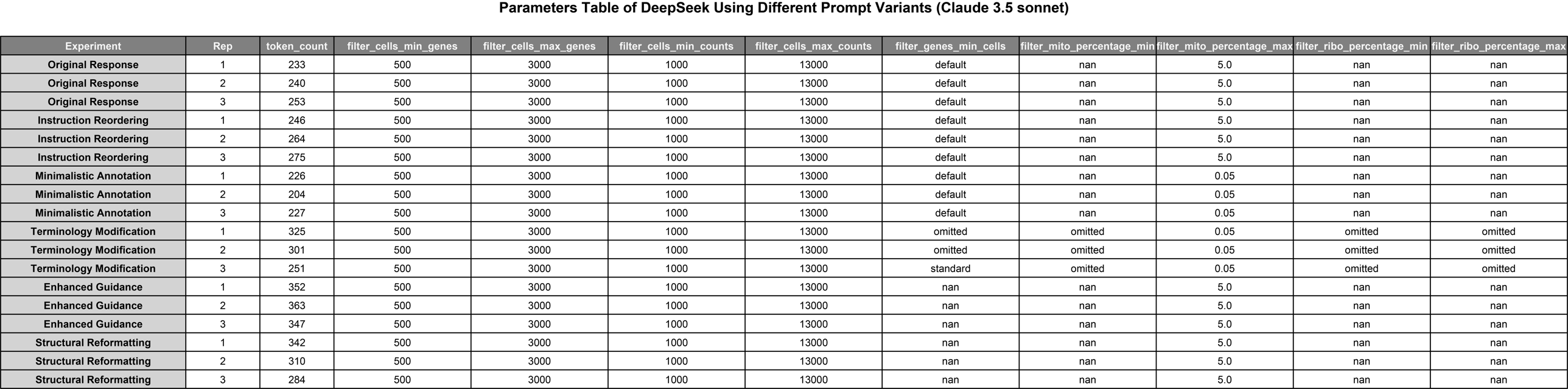


**Fig S10. Impact of prompt variations on parameter extraction and annotation consistency**

(A-B) Tables presents extensive parameter extraction across different prompt variants from (A) Deepseek v3 and (B) Claude 3.5 Sonnet outputs. Each row represents a specific prompt variant (Original, Instruction_Reordering, Terminology_Modification, Enhanced_Guidance, Minimalistic_Annotation, and Structural_Reformatting), with columns indicating replicate number, token count, and extracted parameter values. Parameter extraction remains highly consistent across variants, with only minor differences in terminology ('omitted' vs 'nan') or units (0.05 vs 5 for mitochondrial percentage). Claude 3.5 Sonnet demonstrates superior robustness with simplified prompts compared to Deepseek v3, which occasionally struggles with minimal instruction formats.


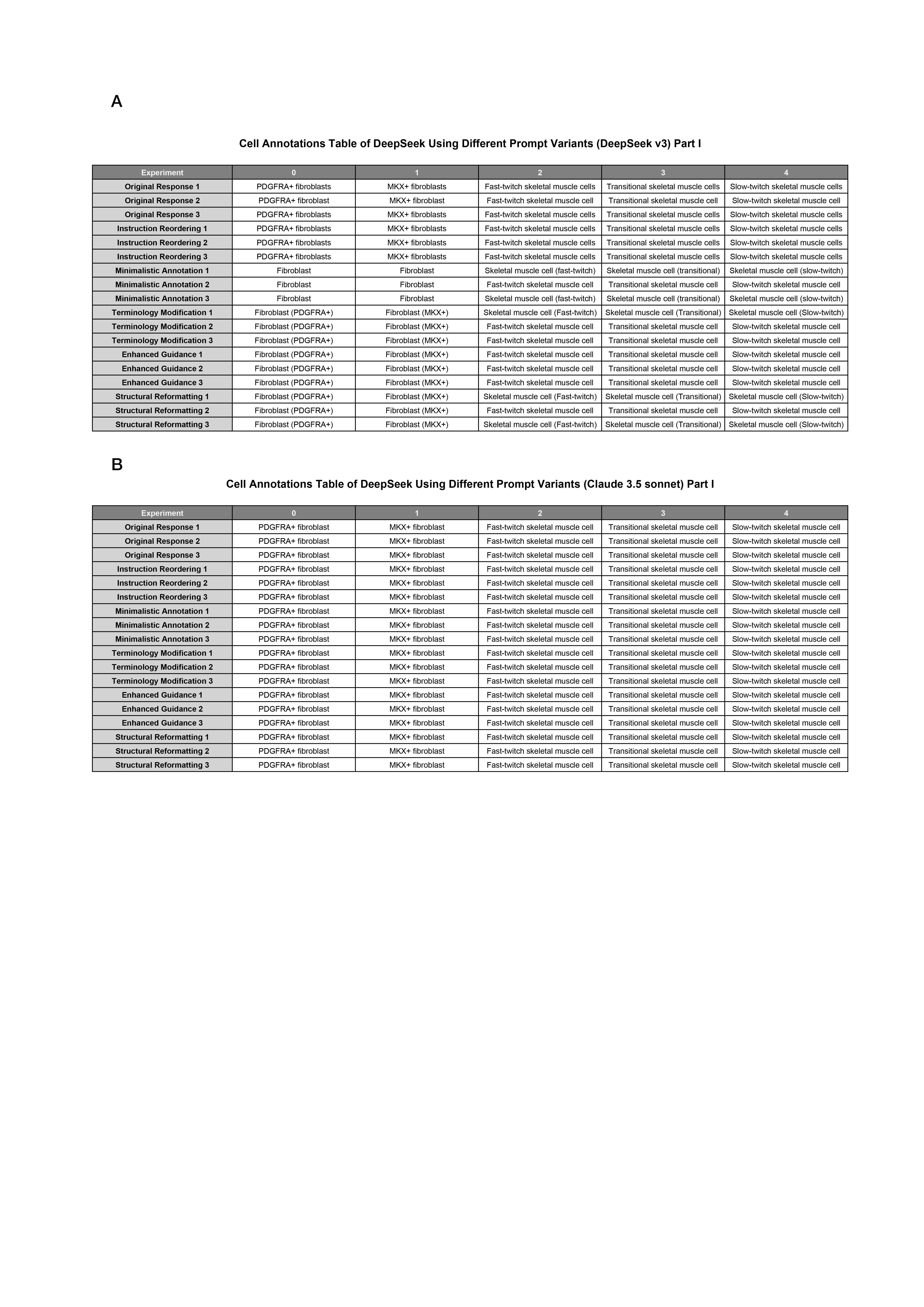


**Fig S11. Evaluation of prompt variation effects on cell type annotation across different LLM models**

(A-B) Tables showing comparative analysis of how different prompt variants affect cell type annotation output using (A) Deepseek v3 and (B) Claude 3.5 Sonnet. Each column represents a different prompt variant (Original, Instruction_Reordering, Terminology_Modification, Enhanced_Guidance, Minimalistic_Annotation, and Structural_Reformatting), while rows correspond to distinct cell clusters. The tables show that annotation content remains largely stable across prompt variants, particularly for well-defined cell types. For Deepseek v3, format variations occur mainly in subtype representation, with some prompts placing subtype information in parentheses. The Minimalistic_Annotation prompt resulted in simplified fibroblast subtype information, while muscle subgroup annotations showed some ambiguity. While Claude 3.5 Sonnet demonstrated superior robustness, maintaining nomenclature closely aligned with the original text across all prompt variants.


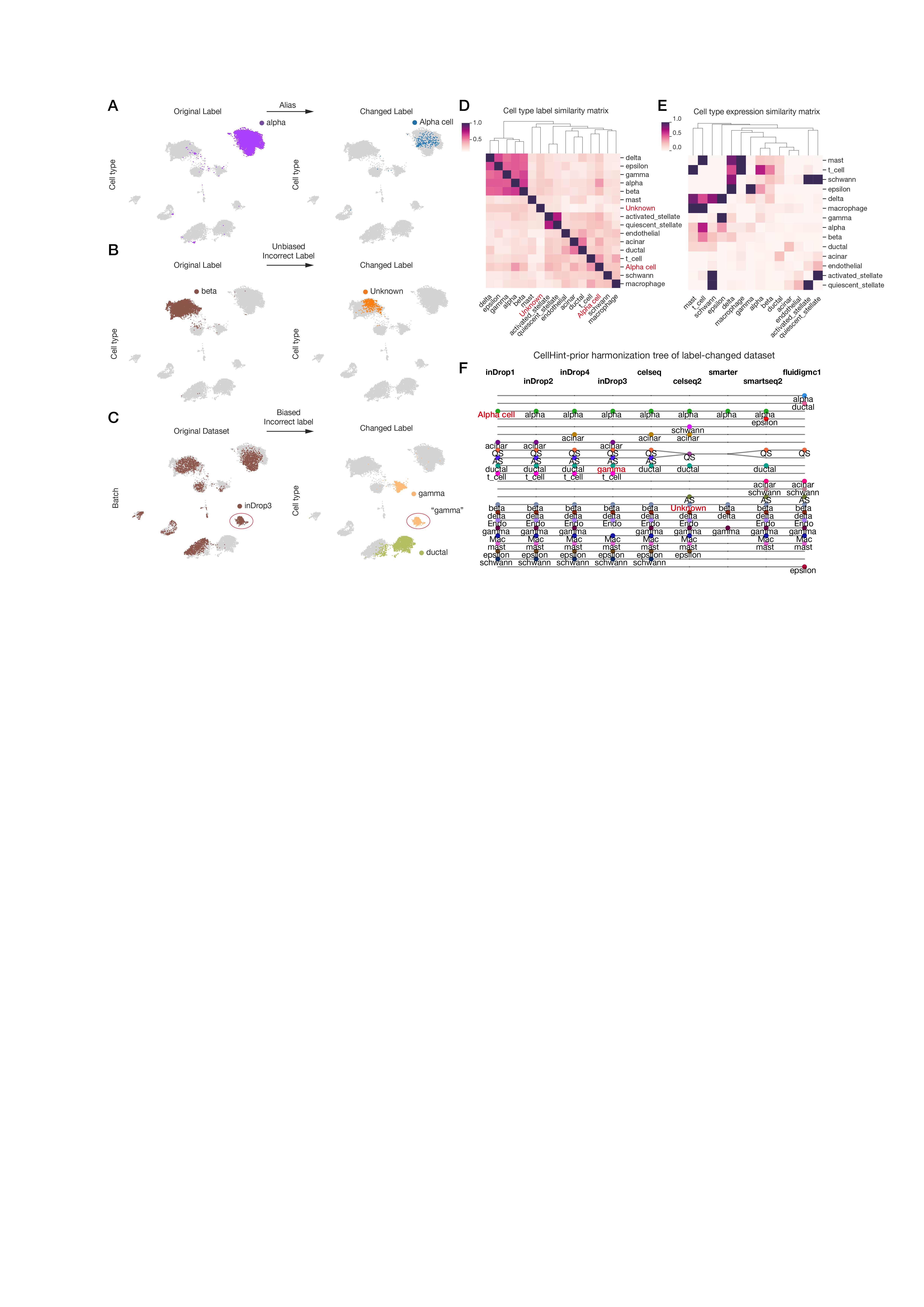


**Fig S12. Analysis of robustness of correction of incorrect labels**

(A-C) UMAP plot showing alias (A), unbiased incorrect label (B) and biased incorrect label change (C) in pancreas dataset.

(D) Matrix plot showing similarities between cell type annotations with embedding generated by OpenAI's text-embedding-3-large model.

(E) Matrix plot showing similarities between cell type expressions.

(F) Tree plot showing cell type harmonization results of cellhint-prior with three types of label change.


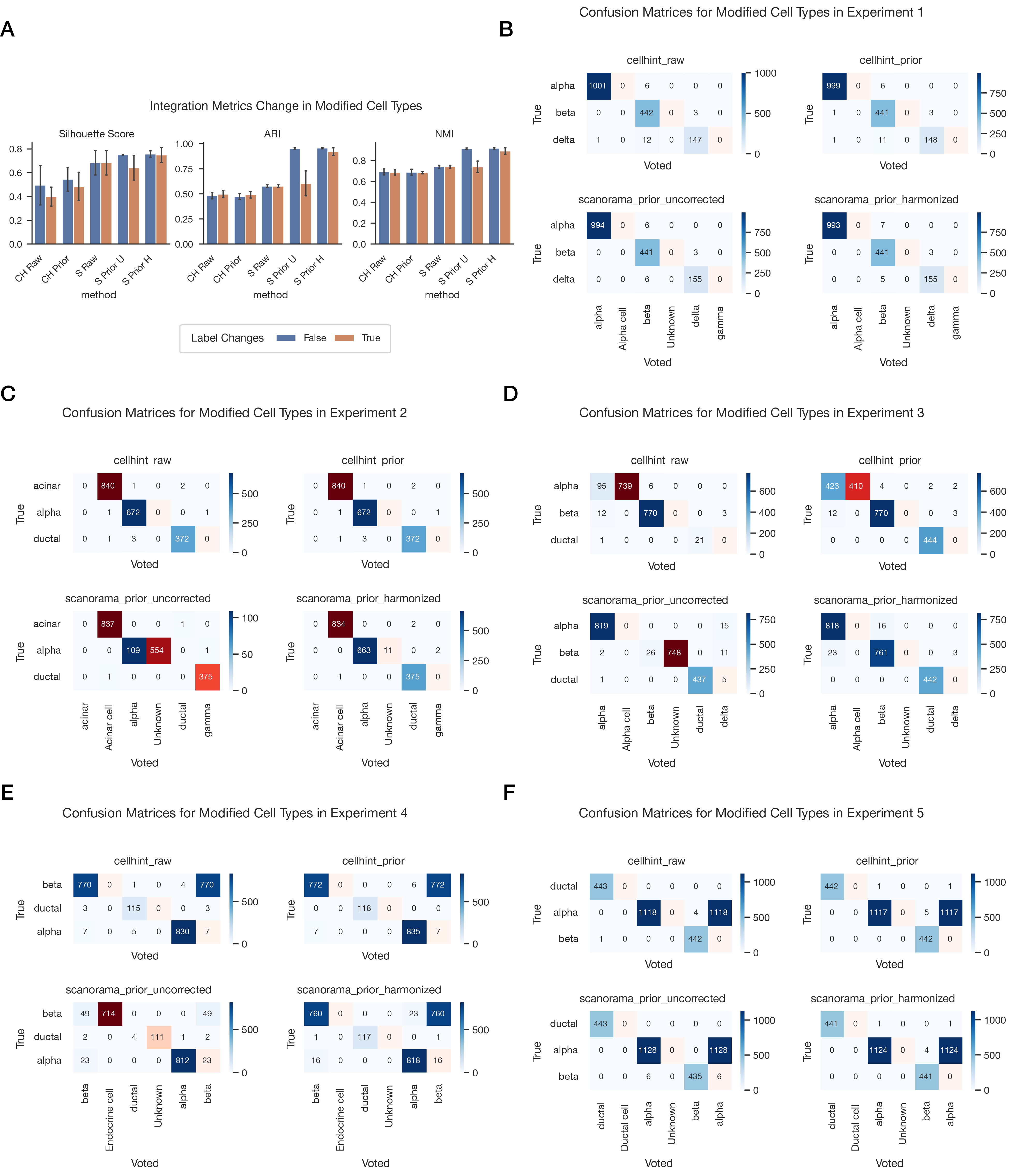


**Fig S13. Systematic evaluation of label change effects across different integration methods**

(A) Bar plot showing changes in integration performance metrics before and after label modification for affected cell types. Metrics from left to right: Silhouette score, Adjusted Rand Index (ARI), and Normalized Mutual Information (NMI).

(B-F) Heatmaps demonstrating changes in major voting-derived cell type classifications compared to ground truth annotations before and after label modification. Red cells indicate misclassifications due to annotation changes, while blue cells represent populations that maintained original cell type labels by clustering with corresponding populations from other datasets despite annotation changes. Integration methods evaluated in each panel: cellhint, cellhint_prior, direct scanorama_prior using annotated cell types, and two-step integration using harmonized cell types for scanorama_prior.


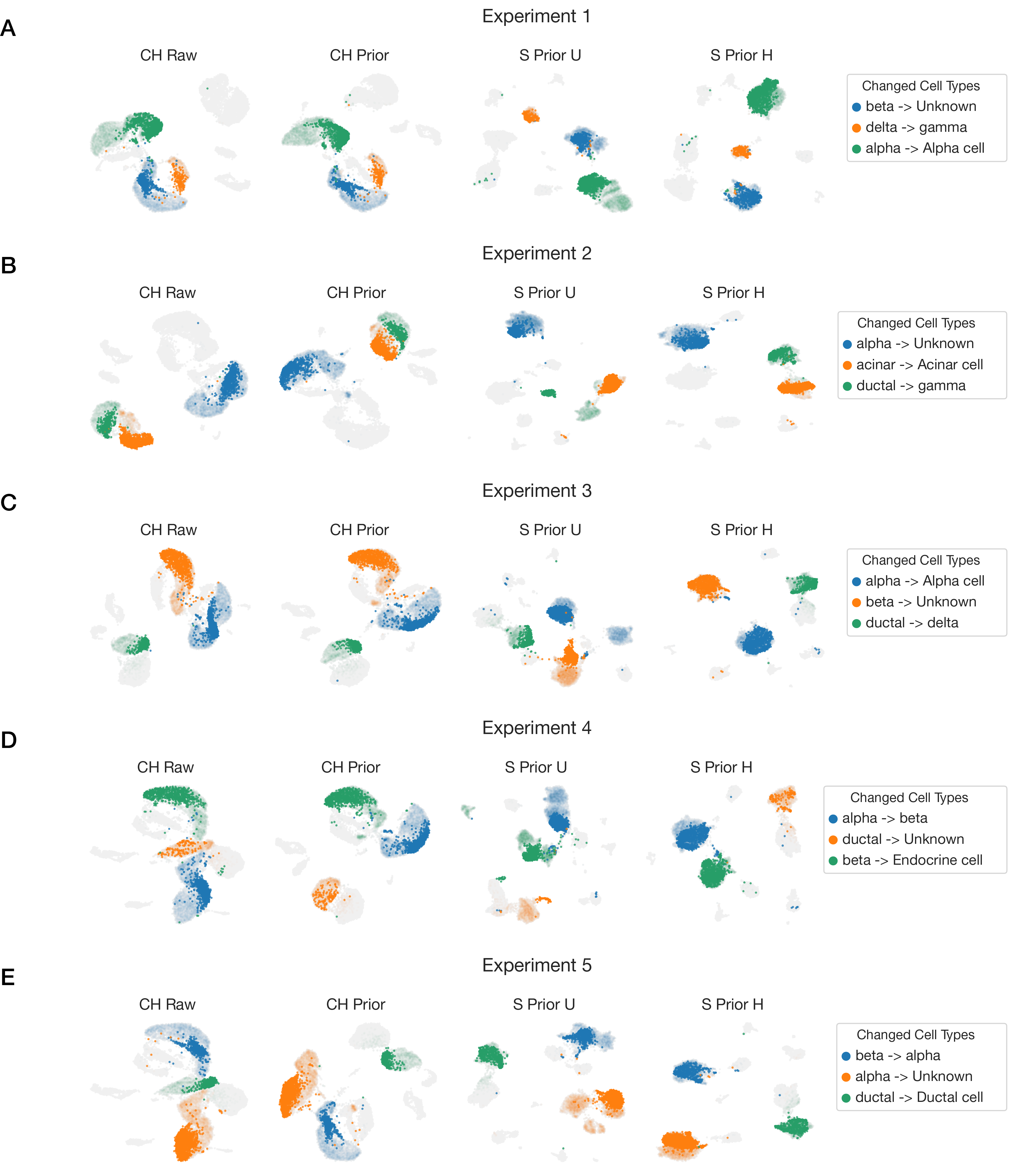


**Fig S14.** **UMAP evaluation of label change effects across different integration methods**

(A-E) UMAP visualizations demonstrating embedding changes before and after label modification experiments. Darker shades represent cells from datasets with modified labels, while lighter shades indicate cells retaining their original cell type annotations. Integration methods from left to right: cellhint, cellhint_prior, direct scanorama_prior using annotated cell types, and two-step integration using harmonized cell types for scanorama_prior.


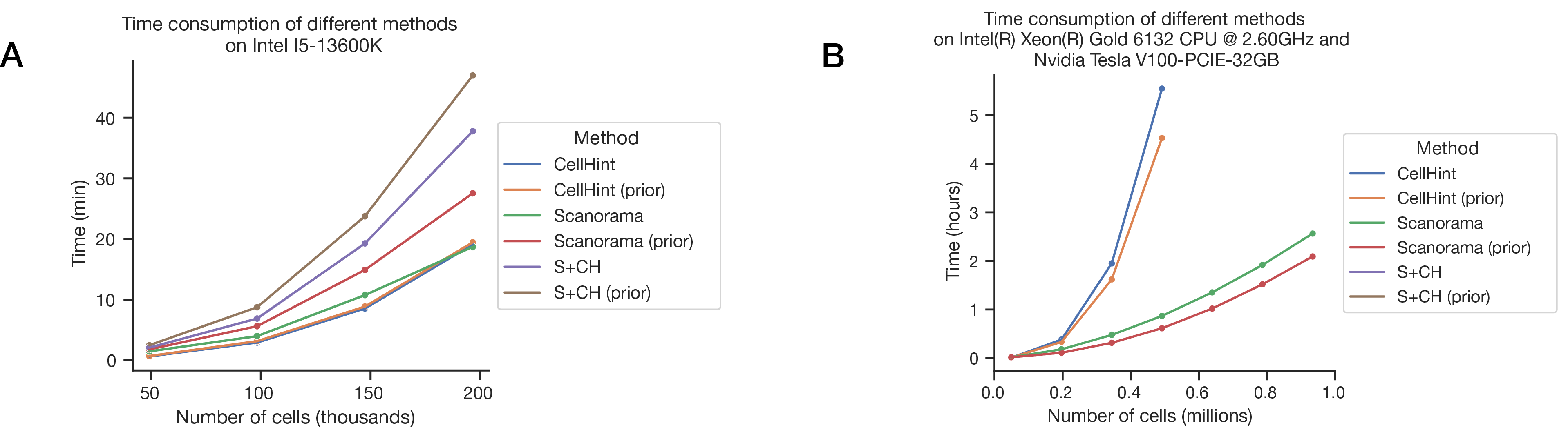


**Fig S15. Computational performance of scExtract integration method**

(A) Runtime scaling analysis of different integration methods with increasing dataset size on a personal computer processor.

(B) Runtime scaling comparison of different integration methods with increasing dataset size under server CPU and GPU acceleration. Cellhint-based methods were excluded from benchmarking for datasets exceeding 500,000 cells due to large computational time requirements.


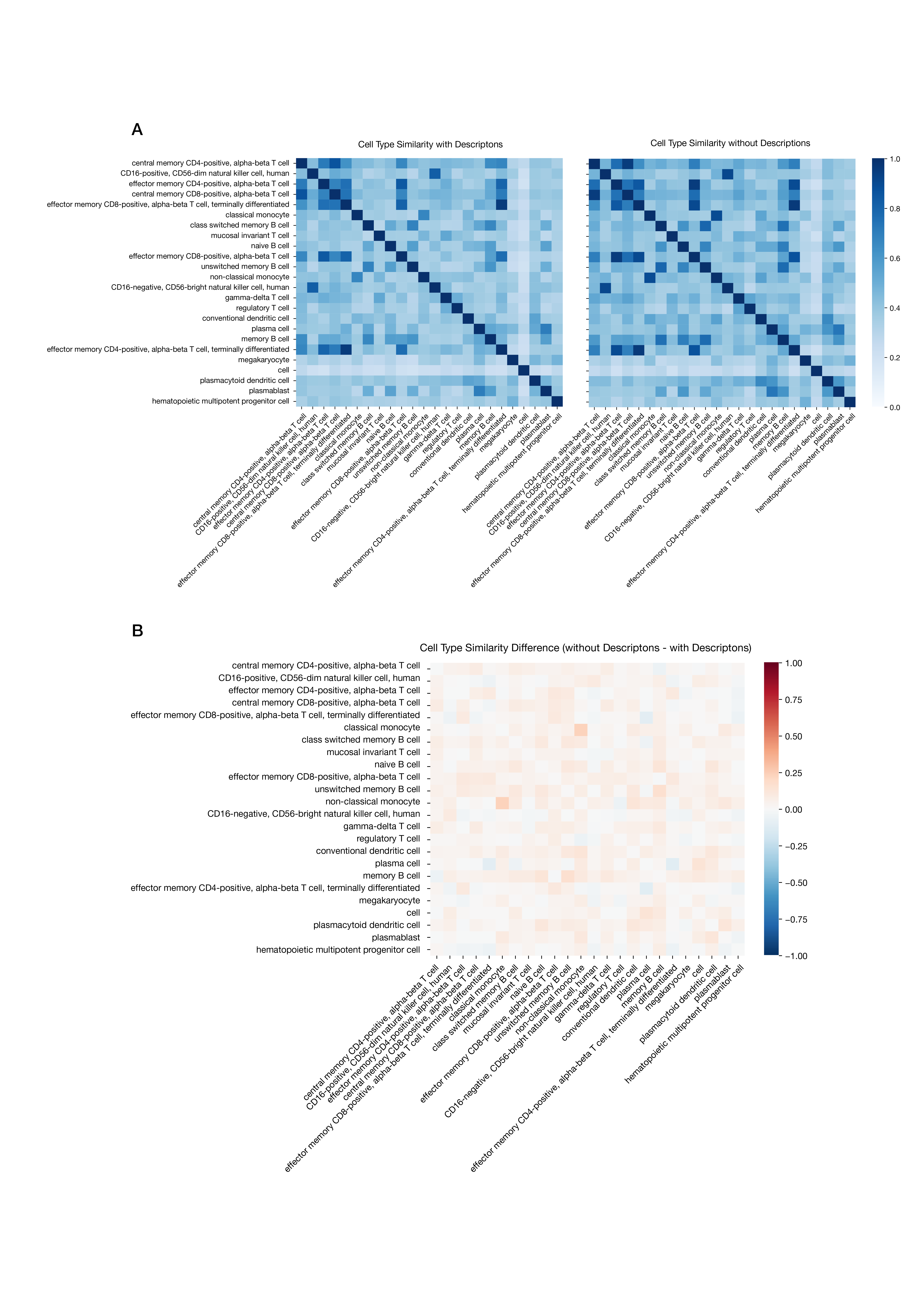


**Fig S16. Cell type similarity matrices with and without descriptive information**

(A) Heatmap displaying similarity matrices between all cell types in the Blood dataset, with the left panel showing similarities using descriptions and the right panel showing similarities without descriptions.

(B) Heatmap displaying the result of subtracting the similarity matrix with descriptions from the similarity matrix without descriptions in the Blood dataset.


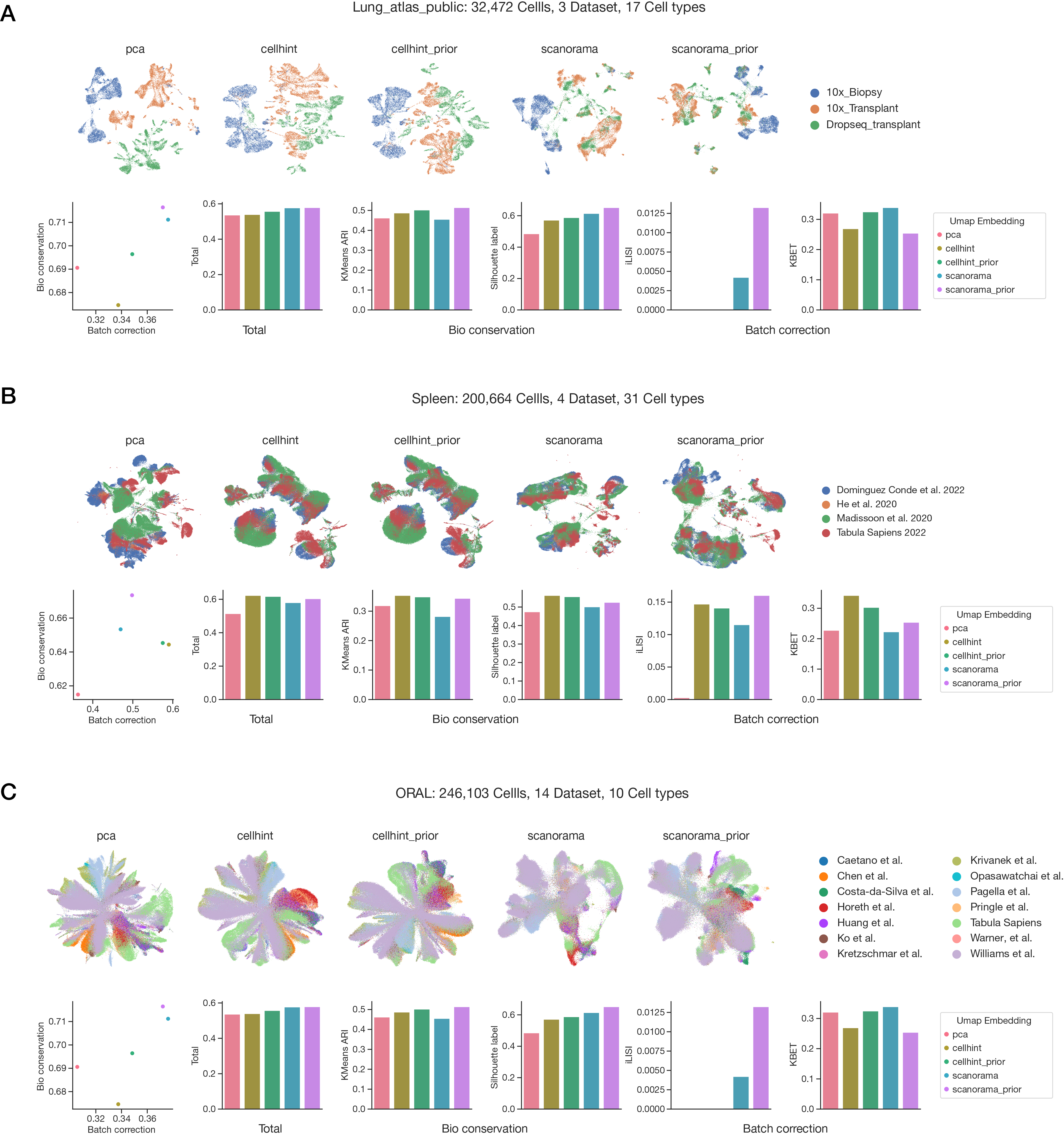


**Fig S17. Assessment of scExtract's two-step integration strategy on large-scale datasets**

(A-C) Systematic evaluation of integration performance on Lung_cell_atlas cells (A), Spleen cells (B), and Human Oral Cell Atlas (C) datasets. Dataset statistics (number of cells, datasets, and cell types) are indicated above each panel. Upper panel: UMAP visualizations of embeddings generated by different methods (from left to right): PCA, cellhint integration, cellhint_prior integration, original scanorama, and scExtract's two-step integration derived scanorama_prior. Lower panel: Performance metrics (from left to right): scatterplot showing batch effect removal versus biological variation preservation, barplot of comprehensive performance metrics, barplots displaying KMeans Adjusted Rand Index (ARI), Silhouette label scores, integration Local Inverse Simpson's Index (iLISI), and k-nearest neighbor Batch Effect Test (kBET).


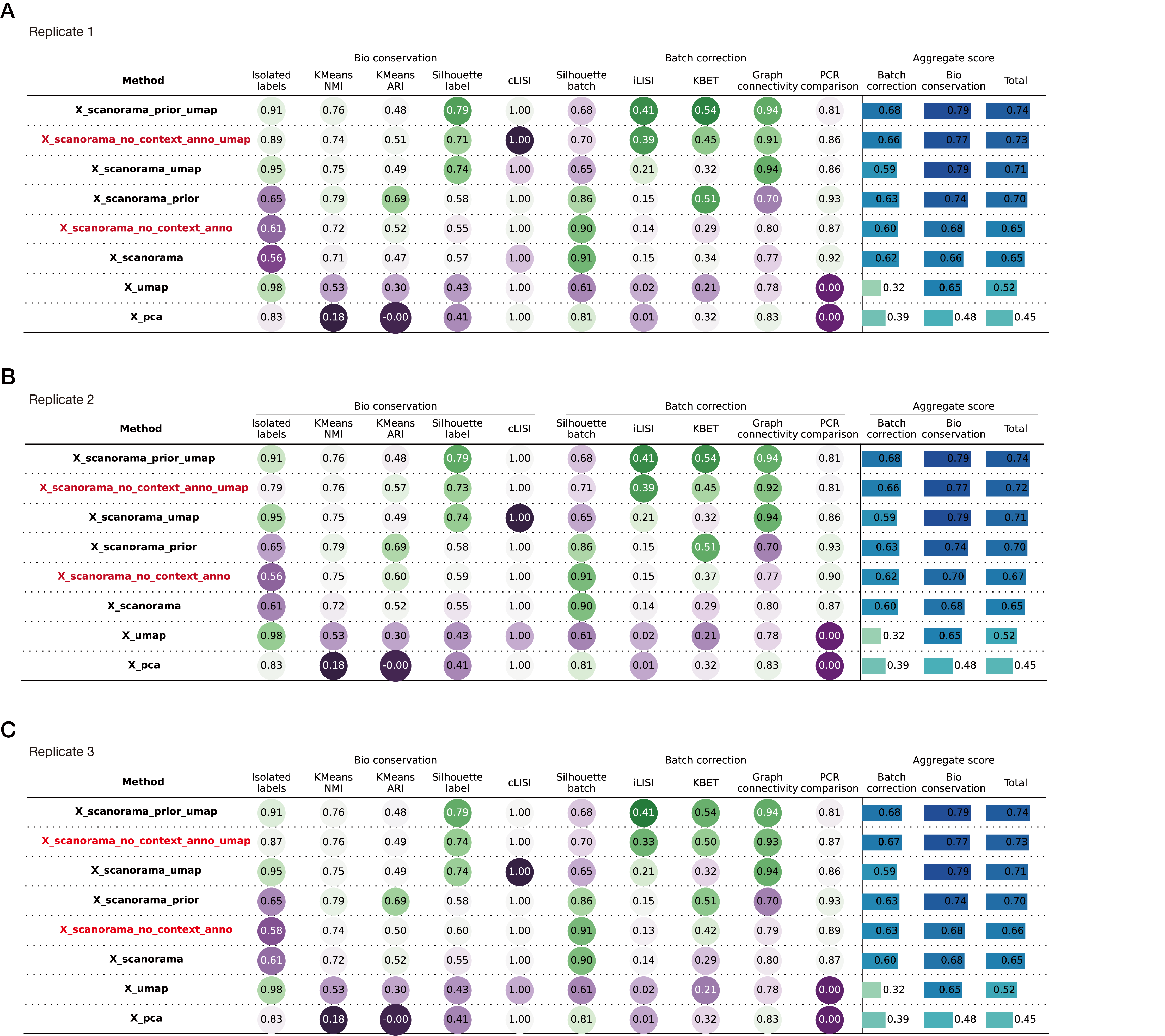


**Fig S18. Performance metrics table for pancreas dataset integration using scExtract (no context) annotation**

(A-C) Performance metrics table for pancreas dataset integration using scib-metrics, where each row represents a different method and each column represents a metric name, with overall performance shown as bar charts on the right side. Each panel represents a replicate.


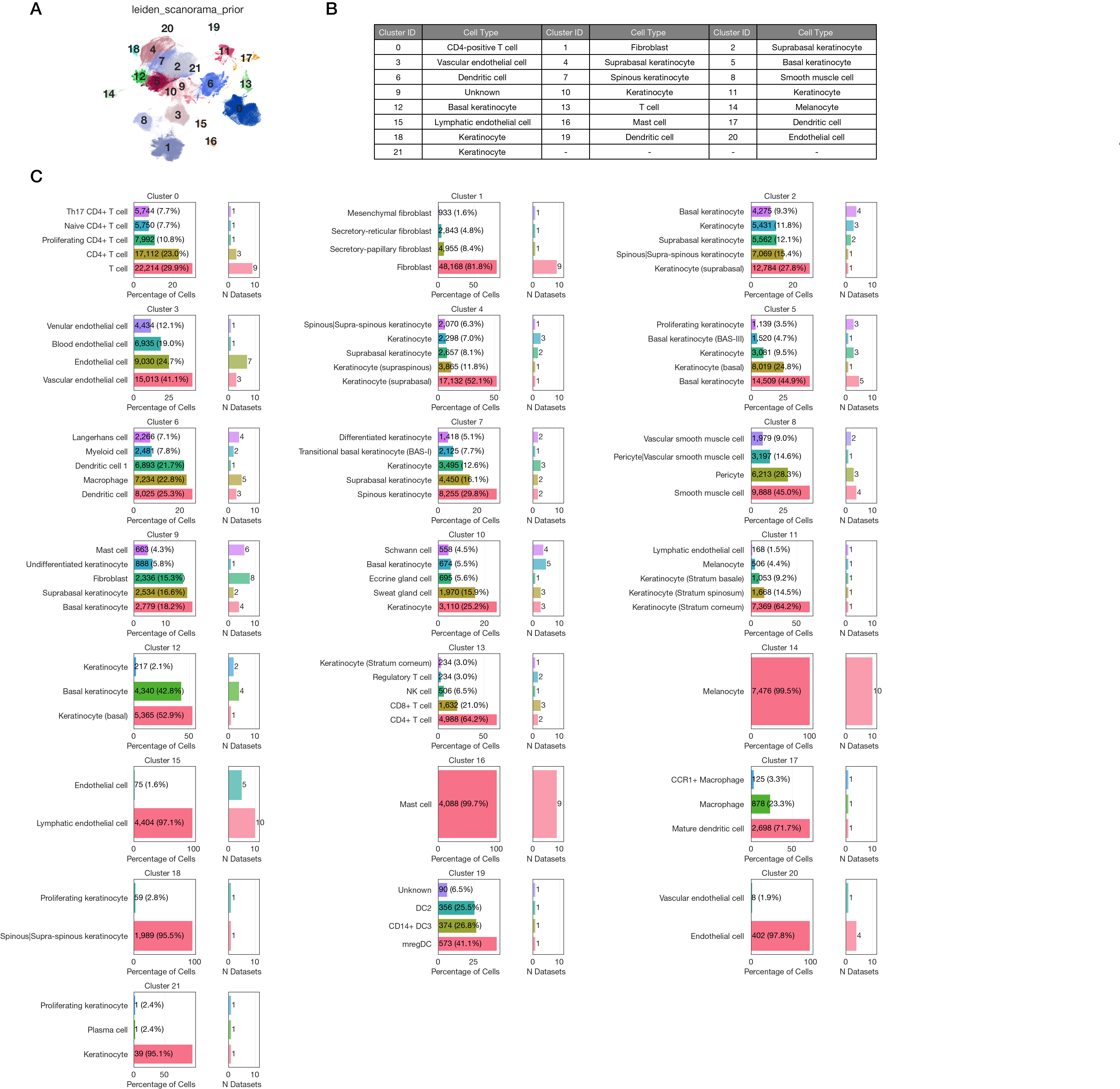


**Fig S19. Major voting-integrated cell type annotations assisted by scExtract**

(A) UMAP visualization showing unsupervised clustering of scExtract integration embeddings.

(B) Table presenting integrated annotation results for each cluster.

(C) Horizontal bar plot displaying the top 5 original annotations for each cluster, showing cell counts, proportions, and the number of source datasets represented.
